# Nexus of Soil Microbiomes, Genes, Classes of Carbon Substrates, and Biotransformation of Fluorotelomer-Based Precursors

**DOI:** 10.1021/acs.est.4c06471

**Published:** 2024-11-06

**Authors:** Jinha Kim, Scott W. Leonard, Mariann Inga Van Meter, Mitchell L. Kim-Fu, Dunping Cao, Jennifer A. Field, Kung-Hui Chu

**Affiliations:** † Zachry Department of Civil and Environmental Engineering, Texas A&M University, College Station, Texas 77843, United States; ‡ Department of Environmental and Molecular Toxicology, Oregon State University, Corvallis, Oregon 97331, United States; § Department of Chemistry, Oregon State University, Corvallis, Oregon 97331, United States

**Keywords:** per- and polyfluoroalkyl substances (PFAS), fluorotelomer, defluorination, cocamidopropyl betaine, alkanesulfonate monooxygenase, 5:3 fluorotelomer carboxylic acid

## Abstract

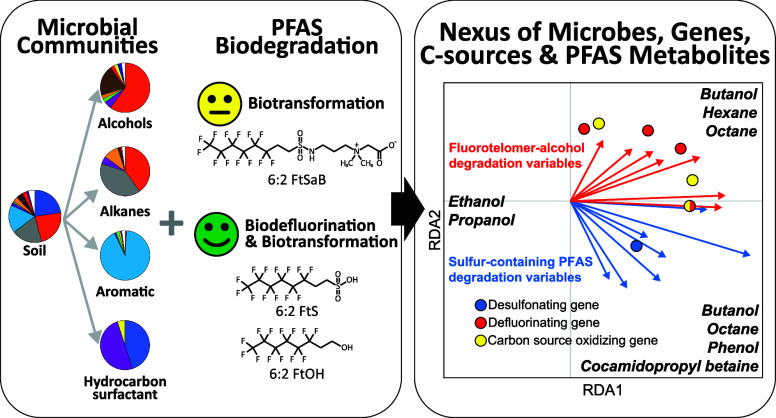

The unpredictable biodegradation of fluorotelomer (FT)-based per- and polyfluoroalkyl substances (PFAS) causes complicated risk management of PFAS-impacted sites. Here, we have successfully used redundancy analysis to link FT-based precursor biodegradation to key microbes and genes of soil microbiomes shaped by different classes of carbon sources: alcohols (C2–C4), alkanes (C6 and C8), an aromatic compound (phenol), or a hydrocarbon surfactant (cocamidopropyl betaine [CPB]). All the enrichments defluorinated fluorotelomer alcohols (*n*:2 FtOH; *n* = 4, 6, 8) effectively and grew on 6:2 fluorotelomer sulfonate (6:2 FtS) as a sulfur source. The butanol-enriched culture showed the highest defluorination extent for FtOHs and 6:2 FtS due to the high microbial diversity and the abundance of desulfonating and defluorinating genes. The CPB-enriched culture accumulated more 5:3 fluorotelomer carboxylic acid, suggesting unique roles of *Variovorax* and *Pseudomonas*. Enhanced 6:2 FtOH defluorination was observed due to a synergism between two enrichments with different carbon source classes except for those with phenol- and CPB-enriched cultures. While the 6:2 fluorotelomer sulfonamidoalkyl betaine was not degraded, trace levels of 6:2 fluorotelomer sulfonamidoalkyl amines were detected. The identified species and genes involved in desulfonation, defluorination, and carbon source metabolism are promising biomarkers for assessing precursor degradation at the sites.

## Abstract 2

Biodefluorination and biotransformation of fluorotelomer-based precursors are controlled by alcohol (C2–C4)-, alkane (C6 & C8)-, phenol-, and cocamidopropyl betaine-utilizing soil microbiomes.

## 1 Introduction

Per- and polyfluoroalkyl substances (PFAS) are man-made compounds that generate high public health concerns since some are toxic, persistent, and widespread in soil, water, food, and biota.^1−4^ Numerous types of PFAS are ubiquitous within indoor environments^5−7^ and in engineered or natural systems ranging from wastewater treatment plants, landfill leachate, surface water, groundwaters, to sediments.^8−13^ Fluorotelomer (FT)-based PFAS such as 6:2 fluorotelomer alcohol (FtOH) and 6:2 fluorotelomer sulfonate (FtS) are widely found in paints, textiles, sprays, and food contact paper.^14−17^ Zwitterionic 6:2 fluorotelomer sulfonamidoalkyl betaine (FtSaB)^18,19^ is a common ingredient in aqueous film forming foams (AFFFs).^20^ Animal studies have shown that 6:2 FtOH and 8:2 FtOH are potential carcinogens^21,22^ and endocrine disruptors that can cause reproductive dysfunction.^23,24^ Bioaccumulation of 6:2 FtSaB, 6:2 FtS, and their metabolites have been reported in mice, fish, humans,^25,26^ and plants.^27^

Recent studies revealed that zwitterionic and cationic PFAS contribute to a significant fraction (up to 97%) of total PFAS.^28^ Furthermore, approximately 52% of the total PFAS are associated with polyfluorinated precursors in firefighter training area soil.^29^ Retention of PFAS precursors in the source zones acts as a long-term source for the transport/transformation of PFAS to downgradient soil, surface water, and biota as well as to groundwater. While biodegradation of 6:2 FtS and 6:2 FtSaB to perfluorocarboxylic acids (PFCA) have been reported,^30−32^ the precursors are persistent in groundwater at inactive military sites for decades.^33^ Thus, gaining insights into how soil microbiomes biodegrade FT-based PFAS precursors under varying conditions is vital for predicting the fate of PFAS and developing effective risk management strategies for highly FT-based PFAS contaminated sites.

Mixed cultures from activated sludge, river sediments, soils, and a limited number of pure strains biotransform FT-based PFAS to varying degrees.^34^ Previous studies on FtOH and 6:2 FtS biotransformation using pure cultures show the importance of carbon types for inducing different enzymes for effective defluorination.^35−38^ For instance, 1-butanol (BuOH)-grown *Pseudomonas butanovora* (now *Thauera butanivorans*) transformed 6:2 FtOH to only PFCAs but transformed to both PFCAs and 5:3 fluorotelomer carboxylic acid (FtCA) with lactate addition.^38^ Octane-grown *Pseudomonas oleovorans* similarly produced PFCAs and 5:3 FtCA.^37^ Octane, ethanol (EtOH), and BuOH amendments to activated sludge cultures facilitated expression of high levels of alkane monooxygenase and were linked to higher fluoride release from 6:2 FtOH and 6:2 polyfluoroalkyl phosphate (PAP).^39,40^ Various 6:2 FtOH degradation patterns by different combinations of known FtOH-degrading microbes were observed.^39^ All of the previous findings suggested that different pools of induced enzymes in degradative strains could exhibit synergistic or inhibitory effects on FtOH degradation patterns. However, no study exploring this concept by combining different microbial communities shaped by various carbon source types has been reported. *Rhodococcus jostii* RHA1 degraded more 6:2 FtS when grown with BuOH than that with EtOH or octane in sulfur (S)-limited conditions.^35,36^ Biodegradation of 6:2 FtS effectively occurs under S-limited conditions as 6:2 FtS can be used as a sole S-source within the time-scale of days.^35,41,42^ In S-rich conditions, 6:2 FtS could be degraded but requires significantly longer time-scale of months to occur.^43^ The longer duration could be because excessive sulfate is being competed against 6:2 FtS and must be exhausted prior to 6:2 FtS desulfonation. Acetate allowed 70% 6:2 FtSaB degradation by *Gordonia* sp. NB4-1Y,^42^ but 6:2 FtSaB remained persistent in activated sludge.^43^ Stability of 6:2 FtSaB is also observed during petroleum hydrocarbon (C10–C50) degradation.^44^ Interestingly, amendment of aromatic compounds such as benzene, toluene, ethylbenzene, and o-xylene (BTEX) increased 6:2 fluorotelomer thioether amido sulfonate (FtTAoS) degradation in soil microcosms.^45^ Despite the promise of carbon amendments to promote precursor biotransformation in mixed cultures, no study has systematically evaluated the effects of various classes of carbon sources on microbial dynamics that link with PFAS degradation. Several genes encoding enzymes involved in the desulfonation of sulfur-containing PFAS^35,41^ and defluorination enzymes of 6:2 FtS and FtOHs^35^ have been confirmed from pure culture studies. However, these genes are seldom assessed in mixed culture studies. Additionally, many non-PFAS constituents such as hydrocarbon surfactants are present in AFFF^46^ and have been recently detected as PFAS cocontaminants in AFFF-impacted matrices.^47^ Hydrocarbon surfactants are a different class of organics than aliphatic and aromatic compounds, which could potentially inhibit microbial growth or serve as a carbon source.^48,49^ However, to the best of our knowledge, no study has examined the impacts of hydrocarbon surfactants on soil microbiomes and precursor biodegradation.

To fill the knowledge gaps described above, we systematically evaluated the biodegradation of FT-based PFAS by soil microbiomes grown on different carbon sources: C2–C4 alcohols (EtOH, 1-propanol [PrOH], and BuOH), C6 and C8 alkanes (hexane and octane), an aromatic compound (phenol), and a hydrocarbon surfactant (cocamidopropyl betaine [CPB]).^50^ In this study, CPB was selected because it is a common hydrocarbon surfactant in AFFF.^46,51,52^ The CPB is a mixture of different chain lengths of alkylamidopropyl betaines with 50% of lauramidopropyl betaine. As the CPB shares a similar betaine structure with 6:2 FtSaB, we hypothesized that CPB-degrading soil microorganisms can also degrade 6:2 FtSaB. All of the enrichment cultures were examined for their ability to degrade various types of FT-based PFAS: *n*:2 FtOH (*n* = 4, 6, and 8), 6:2 FtS, and 6:2 FtSaB under S-rich or S-limiting conditions. Precursor biodegradation was further linked with microbial community dynamics and predicted functional gene profiles that were shaped by carbon sources.

## 2 Materials and Methods

### 2.1 Chemicals

High-purity PFAS standards were purchased from Wellington Laboratories and used for PFAS chemical analysis. Details of PFAS standards (Tables S1 and S2) and other used chemicals are available in the Supporting Information Section S1a. Briefly, 4:2 FtOH (≥97%; CAS # 2043-47-2) was purchased from TCI (Portland, OR). Two FtOHs, 6:2 FtOH (97%; CAS # 647-42-7) and 8:2 FtOH (97%; CAS # 678-39-7), were purchased from Sigma-Aldrich (St. Louis, MO). Two S-containing PFAS, 6:2 FtS (≥97%; CAS # 27619-97-2) and 6:2 FtSaB (97.1%; CAS # 34455-29-3), were purchased from Synquest Laboratories (Alachua, FL) and LGC standards (Teddington, Middlesex, UK), respectively. Unless specified, precursor stock solutions were prepared in 100% methanol (MeOH) and used in biodegradation tests.

### 2.2 Soil and Soil Enrichment Cultures

Clean homogenized background sandy soil collected from a former Air Force base^53^ was used as an inoculum to develop various soil enrichment cultures. The sandy soil, provided by Oregon State University, had an acidic pH of 4.9 and contained 0.05% (w/w) organic matter. Further soil characteristics are listed in Table S3. The soil was used to prepare initial seed enrichments that were subsequently used as an inocula for the operation of quasi-steady-state sequencing batch reactors (SBRs) (Figure S1). All enrichments were grown with respective 0.05% (v/v) carbon sources (EtOH, PrOH, BuOH, hexane, octane, phenol, or CPB) in ammonia mineral salt (AMS) medium.^54,55^ A S-free AMS medium was used during subsequent degradation experiments (Table S4). Stable enrichment cultures in the SBRs were established based on the steady OD_600_ and CO_2_ observed over three solid retention times. The stable enrichment cultures were then used for biodegradation experiments and microbial community analysis. A detailed description of the enrichment process and SBR operations is provided in Supporting Information Section S1b.

### 2.3 Biodegradation of FtOHs by Soil Enrichment Cultures

The ability of the enrichment cultures to biotransform FtOHs was assessed based on resting cell assays. The resting cell suspension of the enrichment culture was prepared by washing the cells once with AMS medium and then resuspending in the AMS medium for experimental use. The resting cell assays were conducted in series of 20 mL glass vials. Each vial containing 2 mL of the cell suspension was first crimp sealed with a silicone septum, followed by injecting one of *n*:2 FtOH (*n* = 4, 6, and 8) stock solutions via a gastight Hamilton syringe (Reno, NV) to bring the initial concentration of 20 mg FtOH/L, as described previously.^37,56^ High concentrations of FtOH were used in the resting cell assays, so that fluoride release could be easily detected. The vials were incubated at room temperature with shaking at 160 rpm for 3 days. The liquid samples were then collected for fluoride measurements using a fluoride ion-selective probe.^56^

An additional set of experiments was conducted to assess the synergetic effects of dual enrichments on FtOH biodegradation. In the dual culture experiment, we hypothesized that microbial communities shaped by different carbon sources may interact with each other, synergistically or competitively. These interactions would thus positively or negatively affect the overall FtOH biotransformation. Four enrichment cultures (BuOH-, octane-, phenol-, and CPB-enriched) showing the top four highest fluoride releases from the FtOH biodegradation by the resting cell assays described above were used. The resting cell assays with dual enrichment cultures were set up similarly as described above, except that 1 mL each of two selected enrichments were combined and 6:2 FtOH were used. All experiments were conducted in duplicate.

### 2.4 Biodegradation of 6:2 FtS and 6:2 FtSaB by Soil Enrichment Cultures under S-Limited Conditions

The growth experiments were conducted in a series of glass vials containing 6:2 FtS as the sole S-source in the S-free AMS medium. The experiments were set up as follows. First, the 1 g/L 6:2 FtS stock solution was spiked into a clean 20 mL glass vial. The vial was left open for 20 min to allow MeOH evaporation. Enrichment cultures collected from the SBR were centrifuged, pelleted, and then resuspended with S-free AMS media to achieve a cell suspension of OD_600_ = 0.05. Then, 1 mL of the suspension was allocated into a vial, which was then amended with 6:2 FtS concentration (an initial concentration of 20 mg/L). The vial was crimp-sealed immediately with a silicone septum and spiked with the corresponding carbon sources to initiate the experiment. The vial was incubated at room temperature with shaking at 160 rpm for 8 days before being sacrificed for the fluoride measurement. A parallel set of growth tests with an initial 6:2 FtS concentration of 5 mg/L was conducted to determine metabolites from 6:2 FtS biodegradation by using targeted PFAS analysis. The 6:2 FtSaB growth experiment was slightly modified. Glass vials were replaced with HDPE vials. Two groups of sets were established in which the group 1 set had only an initial carbon spike and incubated for 7 days while the group 2 set was incubated for 9 days while receiving additional carbon spikes of BuOH, phenol, octane, and CPB. Only BuOH-, octane-, phenol-, and CPB-enriched cultures were used. Additional setup information is provided in the Supporting Information Section S1c.

### 2.5 PFAS Analysis and Fluoride Measurement

All liquid samples were mixed with acetonitrile (1:1 v/v) and incubated for 3 days, as described previously.^37,56^ The diluted sample was used for the fluoride measurement using a fluoride ion ion-selective probe (Thermo Scientific, USA).^35^ The detection limit of fluoride using the probe is 20 μg/L, and the linear range of 80 μg/L ∼10 mg/L (*R*^2^ = 0.996–0.999) was used for all fluoride measurements. For PFAS analysis, the diluted sample was first cleaned with 30 mg of ENVI-Carb (Sigma-Aldrich, USA) in a polypropylene (PP) tube, followed by centrifugation (13,000 rpm for 2 min). The clean supernatant was collected and stored in PP vials at 4 °C prior to PFAS analysis. The cleaned samples were then analyzed for PFAS using liquid chromatography-quadrupole time-of-flight mass spectrometry (LC-QToF). Target PFAS analysis was performed for all metabolites and precursors, except FtOHs. For the 6:2 FtSaB degradation samples, both quantitative and qualitative measurements were conducted by using target- and suspect-screening methods, respectively. Detailed LC-QToF operation for PFAS analysis is described in the Supporting Information Section S1d.

### 2.6 Microbial Community Characterization

The collected stable enrichment cultures from the SBRs were characterized, as described previously.^36,57,58^ Detailed microbial community analysis process is given in Supporting Information Section S1e.

## 3 Results and Discussion

### 3.1 Biotransformation of Fluorotelomer Alcohols by Enrichment Cultures

#### 3.1.1 Different Enrichment Cultures Defluorinated and Biotransformed FtOHs to Various Extents

**Figure 1:**
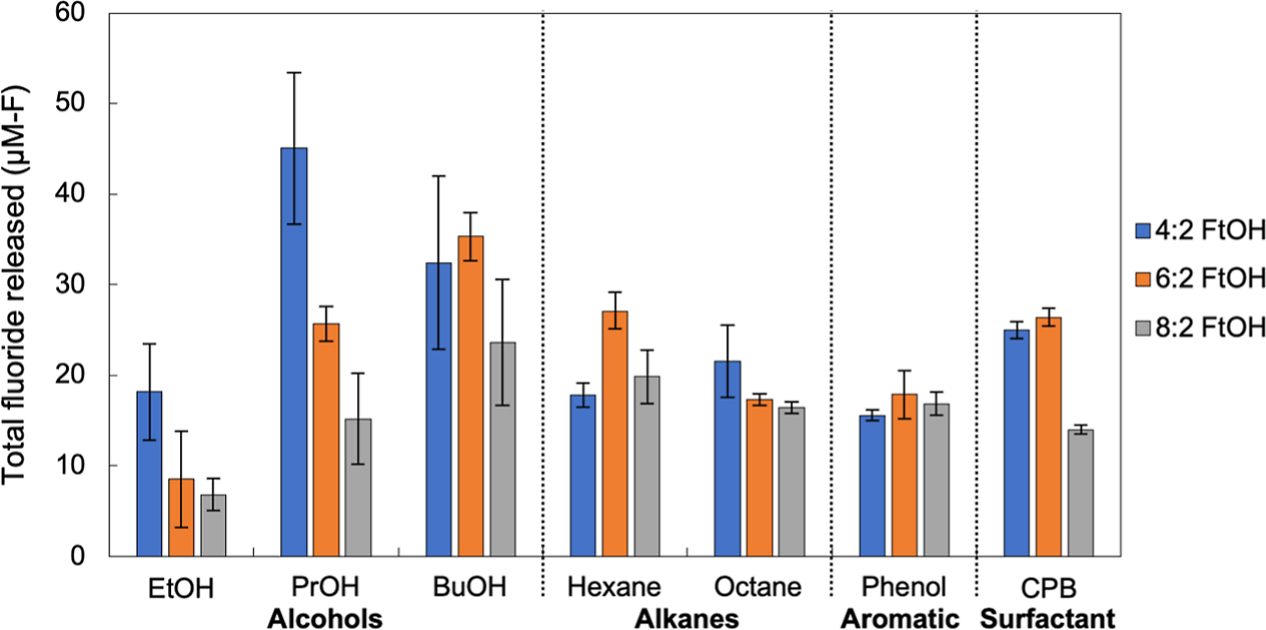
Fluoride from FtOH biodegradation by cultures enriched with different classes of carbon sources. Each vial containing 2 mL of working volume was spiked with 40 μg of FtOH (4:2 FtOH, or 6:2 FtOH, or 8:2 FtOH). The initial FtOH concentrations in vials were 4:2 FtOH (75.5 μM), 6:2 FtOH (55 μM), or 8:2 FtOH (43 μM). Bars represent ranges of duplicate sample measurements.

Among the three alcohol-enriched cultures, the EtOH-enriched culture showed the least fluoride release from all of the FtOHs evaluated (Figure 1). The PrOH-enriched culture showed the highest fluoride concentrations released from 4:2 FtOH (45 ± 6 μM-F), followed from 6:2 FtOH (25.5 ± 2.0 μM-F) and 8:2 FtOH (15 ± 5 μM-F). The BuOH-enriched culture showed the highest fluoride release from 6:2 and 8:2 FtOHs (35.5 ± 2.0 μM-F from 6:2 FtOH; and 23.5 ± 5.0 μM-F from 8:2 FtOH). When comparing the fluoride released from 6:2 FtOH by three alcohol-enriched cultures, a trend was observed with increasing fluoride concentrations, from low to high, by EtOH- < PrOH- < BuOH-enriched cultures. This trend was also observed for 8:2 FtOH by the three alcohol-enriched cultures, suggesting that higher defluorination of longer-chain FtOH appeared to be linked to the enrichment cultures receiving longer-chain alcohols. Additional discussion is provided in Supporting Information Section S2.

No FtOH degradation metabolite was detected in the controls that contained only FtOHs and media. Metabolites of FtOHs were detected only in samples receiving FtOHs and cultures. The metabolite profiles shown in Figure S2 suggested that (i) short-chain alcohol, PrOH, was an effective carbon source for enriching mixed cultures to give a higher defluorination extent (i.e., summation of fluoride equivalent [μM-F] of each of the FtOH metabolites and released fluoride) from 4:2 FtOH degradation, (ii) the BuOH-, hexane-, and octane-enriched cultures showed higher defluorination extent of 6:2 FtOH and 8:2 FtOH, and (iii) the CPB-enriched culture showed an ability to produce 5:3 FtCA from 6:2 FtOH biodegradation. A trace amount of 5:3 FtCA was detected in the 6:2 FtOH degradation experiments using the mono phenol-enriched culture (Figure S2B). Production of 5:3 FtCA is favorable since 5:3 FtCA can be further degraded into shorter-chain PFAS via the one-carbon removal pathway.^59^ In fact, production of 5:3 FtCA from 6:2 FtOH has been observed by mixed and pure culture studies,^37,38,59^ and in soil microcosms,^56^ amended with different carbon sources, such as butanol, octane, sodium fluoroacetate, and lactate. Biodegradation of 5:3 FtCA via one-carbon removal has been demonstrated by a fluoroacetate-degrading bacterium (*Pseudomonas fluorescens* DSM 8341).^38^ However, the kinetics was slow, taking several months to note the degradation of 5:3 FtCA to downstream metabolites.^38^ In this study, we demonstrated that not only BuOH and octane but also hexane, phenol, and CPB are carbon sources that enable 5:3 FtCA production from 6:2 FtOH within 3 days.

#### 3.1.2 Dual Enrichment Cultures Enhanced Biodefluorination and Biotransformation of 6:2 FtOH

Assuming there are no synergistic effects on the defluorination of 6:2 FtOH, the expected fluoride release by the dual cultures is 50% of the summation of fluoride release by the two individual cultures, as measured in Figure 1. All the measured fluoride from the dual enrichments were higher than the averaged released fluoride from the previously assessed mono enrichment cultures by 9.7–36.8%. However, one combination of phenol- and BuOH-enriched cultures gave 37.3% less with respect to the averaged fluoride release (Figure S3). All other combinations with BuOH- and/or octane-enrichment cultures showed an increase in the released fluoride content ranging from 40% to 58% compared to the nonsynergistic released fluoride. The results suggested that multiple carbon substrates, particularly with BuOH and octane, can be applied together to promote defluorinating microbes for effective 6:2 FtOH defluorination at sites. Additional discussion is provided in Supporting Information Section S3.

The metabolites and fluoride produced from 6:2 FtOH by the dual enrichments were plotted along with those by four mono enrichment cultures in Figure S4. By using the defluorination extent concept described above, the defluorination extents of 6:2 FtOH by the dual enrichment cultures were all higher than those of the mono enrichment cultures. The results supported our hypothesis that the synergy between two mono enrichment cultures can enhance 6:2 FtOH biodegradation, except for the combination of phenol- and CPB-enriched cultures. The reason that the combination of phenol- and CPB-enriched cultures was less effective for 6:2 FtOH biodegradation was unclear. However, it might be explained by their unique microbial community and potential gene profiles described later in Sections 3.4 and 3.5. Another interesting observation was the lack of production of 5:3 FtCA in the combination of the phenol- and BuOH-enriched cultures (Figure S4). The lack of 5:3 FtCA production may be explained by the competition between the microbes capable of converting 6:2 fluorotelomer unsaturated carboxylic acid (FtUCA) to 5:3 FtCA and those capable of converting 6:2 FtUCA to 5:2 ketone or 5:2 secondary FtOH in the dual enrichment cultures. Yet, more studies are needed to verify this speculation. Furthermore, novel fluorotelomer-based PFAS degradation metabolites,^60,61^ transformation processes such as CoA adduct formation,^62,63^ conjugation,^64^ and integration into cellular biomass compartments^65^ are being recently discovered. In our study, we observed a lack of production of 5:3 FtCA in the combination of the phenol- and BuOH-enriched cultures, suggesting a potential unknown sink of 5:3 FtCA. Future studies are needed to investigate whether 5:3 FtCA might have entered conjugation or other metabolic pathways^62−64^ in the combination of the phenol- and BuOH-enriched cultures.

### 3.2 Biodegradation of 6:2 FtS and 6:2 FtSaB by Enrichment Cultures

#### 3.2.1 Biodegradation of 6:2 FtS by All Enrichment Cultures

**Figure 2:**
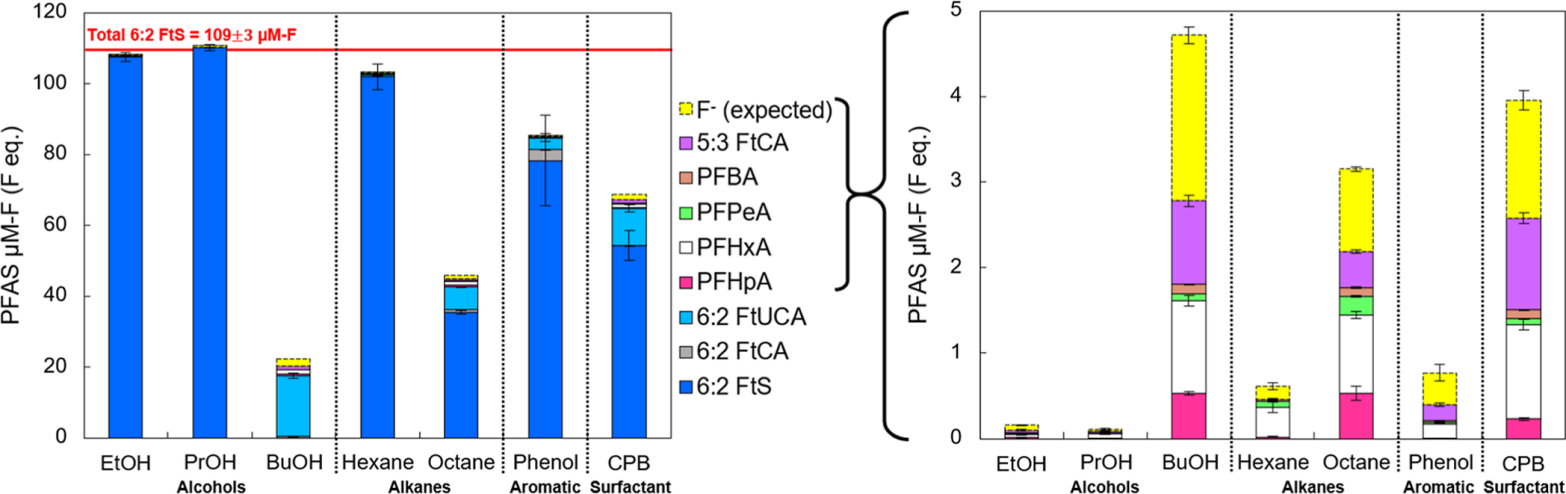
Mass balances based on fluoride equivalents of remaining 6:2 FtS, metabolites, and released fluoride (expected) from the 6:2 FtS degradation experiments in S-free AMS media after 8 days. The 6:2 FtS and media only controls had 6:2 FtS concentration of 109.2 ± 3.1 μM-F (F eq) as measured on days 0, 4, and 8. The right graph shows zoomed-in profile of only perfluoroheptanoic acid (PFHpA), perfluorohexanoic acid (PFHxA), perfluoropentanoic acid (PFPeA), perfluorobutanoic acid (PFBA), 5:3 FtCA, and the released fluoride (expected). There were no measurements of fluoride in the spent growth media. Instead, the expected fluoride release was calculated based the summation of changes of fluoride equivalents between 6:2 FtS and metabolites. For example, as 6:2 FtUCA (12 F eq) is one F eq less than 6:2 FtS (13 F eq), one F eq was expected to release into the medium per 6:2 FtUCA eq being detected. Similarly, detection of one PFHxA (11 F eq) contributes to two F eq and detection of PFHpA (13 F eq) contributes to no fluoride release. Bars represent ranges of duplicate sample measurements.

All the enrichment cultures were able to grow in S-free AMS medium containing only 6:2 FtS (initial 6:2 FtS concentration of 20 mg/L) as a S-source within 8 days. We noticed slight growth of cultures in S-free AMS medium without 6:2 FtS, resulting from the trace sulfur as the impurities of chemicals that were used for preparing the S-free AMS medium (Table S4). Different amounts of fluoride were detected in all of the enrichment cultures, with the highest fluoride mass in the BuOH-enriched culture and the lowest fluoride mass in the hexane-enriched culture (Figure S5). For the repeated growth experiments with a lower initial 6:2 FtS concentration (5 mg/L), different 6:2 FtS removals and various metabolite production were observed (Figure 2). Removals of 6:2 FtS, from high to low, were observed by enrichments grown with BuOH (99.9 ± 0.002%) > octane (67.5 ± 0.4%) > CPB (50.3 ± 3.9%) > phenol (28.3 ± 11.7%) > hexane (6.7 ± 3.3%) > PrOH (1.9 ± 0.8%) > EtOH (1.7 ± 1.1%). Diverse metabolites were detected by BuOH-, octane-, and CPB-enriched cultures with 6:2 FtUCA as the dominant metabolite (Figure 2). Accumulations of 5:3 FtCA were observed in BuOH-, octane-, and CPB-enriched cultures, consistent with observations from the 6:2 FtOH degradation experiments. However, different from 6:2 FtOH degradation, poor 6:2 FtS removal was observed by the hexane-enriched culture. Another exception was the phenol-enriched culture. The sulfur requirement to support microbial growth triggers the desulfonation of 6:2 FtS, followed by defluorination when defluorination enzymes were also expressed in the grown microbes.^35,36^ Given that the sulfur requirement for cell growth is relatively small, it is not surprising to observe persistence of 6:2 FtS in environments containing high sulfur contents. Previous studies have observed persistence of 6:2 FtS in AFFF-impacted soil,^66^ activated sludge,^43,67^ landfill soils,^11^ and wetland soil.^68^ Our previous study^35^ and a recent work^60^ have shown that 6:2 FtS biodegradation were possible when the available sulfur source was depleted via repeat additions of the carbon source to promote cell growth. Results of this current study also implicate that it is possible to accelerate depletion of an available sulfur source in the impacted matrices. Growth of microbes enabling desulfonating and defluorination of 6:2 FtS could be promoted through the addition of trace carbon sources such as BuOH or the exposure to hexane, octane, or CPB in gasoline- or AFFF-impacted sites (Figures 2 and S5).

#### 3.2.2 Biodegradation of 6:2 FtSaB by Four Enrichment Cultures

**Figure 3:**
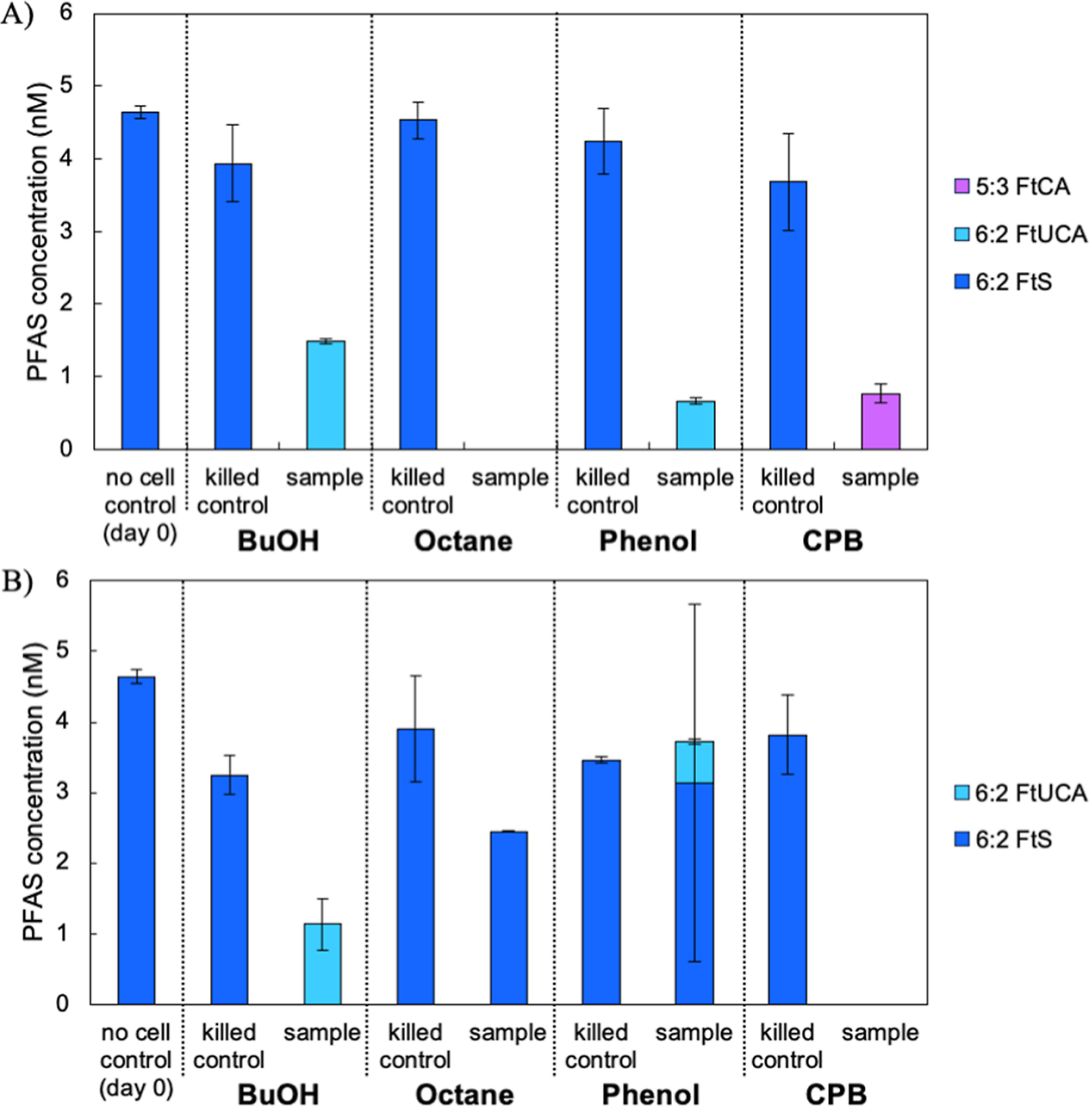
Occurrence of 6:2 FtS transformation products from the 6:2 FtSaB degradation experiments in S-free AMS media by BuOH-, phenol-, octane-, and CPB-enriched cultures. The PFAS concentrations in killed-cell controls and biodegradation samples (A) with additional carbon source amendment at day 9 and (B) without additional carbon source amendment at day 7 are shown. For all conditions, initial carbon sources were spiked on day 0. However, in case for additional carbon source spiking, BuOH, phenol, and CPB were again spiked at days 3 and 7. Octane was spiked on day 5. Bars represent ranges of duplicate sample measurements.

The 6:2 FtSaB biodegradation tests using four enrichment cultures were also conducted under S-limited conditions.^35,36,41,69^ No significant (unpaired *t*-test; *p*-value >0.05) 6:2FtSaB degradation by the BuOH-, phenol-, CPB-, and octane-enriched cultures was observed from target-screening (Figures S6 and S7). The results were relatively consistent through suspect screening (Figures S8 and S9). Instead, the trace 6:2 FtS (as impurities) in all samples were completely removed on day 9, with 6:2 FtUCA detected by 1-BuOH- and phenol-enriched cultures and 5:3 FtCA only detected by the CPB-enriched culture (Figure 3). Similar to 6:2 FtOH or 6:2 FtS degradation experiments, the CPB-enriched culture again accumulated 5:3 FtCA. The additional spike of carbon source with two additional days of incubation led to complete degradation of 6:2 FtS in butanol- and CPB-enriched cultures (Figure 3A). However, 6:2 FtS and metabolites remained on day 7 in phenol- and octane-enriched cultures (Figure 3B). Low levels of 6:2 FtS were observed on day 7 in phenol- and octane-enriched cultures.

Based on the suspect-screening method, CPB- and octane-enriched cultures showed a decrease in the signal area of suspect NIST ID #881 (structure of 6:2 fluorotelomer sulfonamidoalkyl amine [FtSaAm] [tertiary amine]) (Figure S10). Impurities of 6:2 FtSaAm (tertiary amine) have also been previously reported in 6:2 FtSaB degradation experiments by wetland soil and activated sludge which also reported trace 6:2 FtS present from day 0.^61,64^ From our study, suspect NIST ID #3825 (structure of 6:2 FtSaAm [secondary amine]), which is commonly reported as a degradation product from 6:2 FtSaAm (tertiary amine), increased correspondingly in the octane-enriched culture (Figure S11).^37,43,70^ Further information is provided in Supporting Information Section S4.

### 3.3 Carbon Source Type Modulates Unique Microbial Community Dynamics

**Figure 4:**
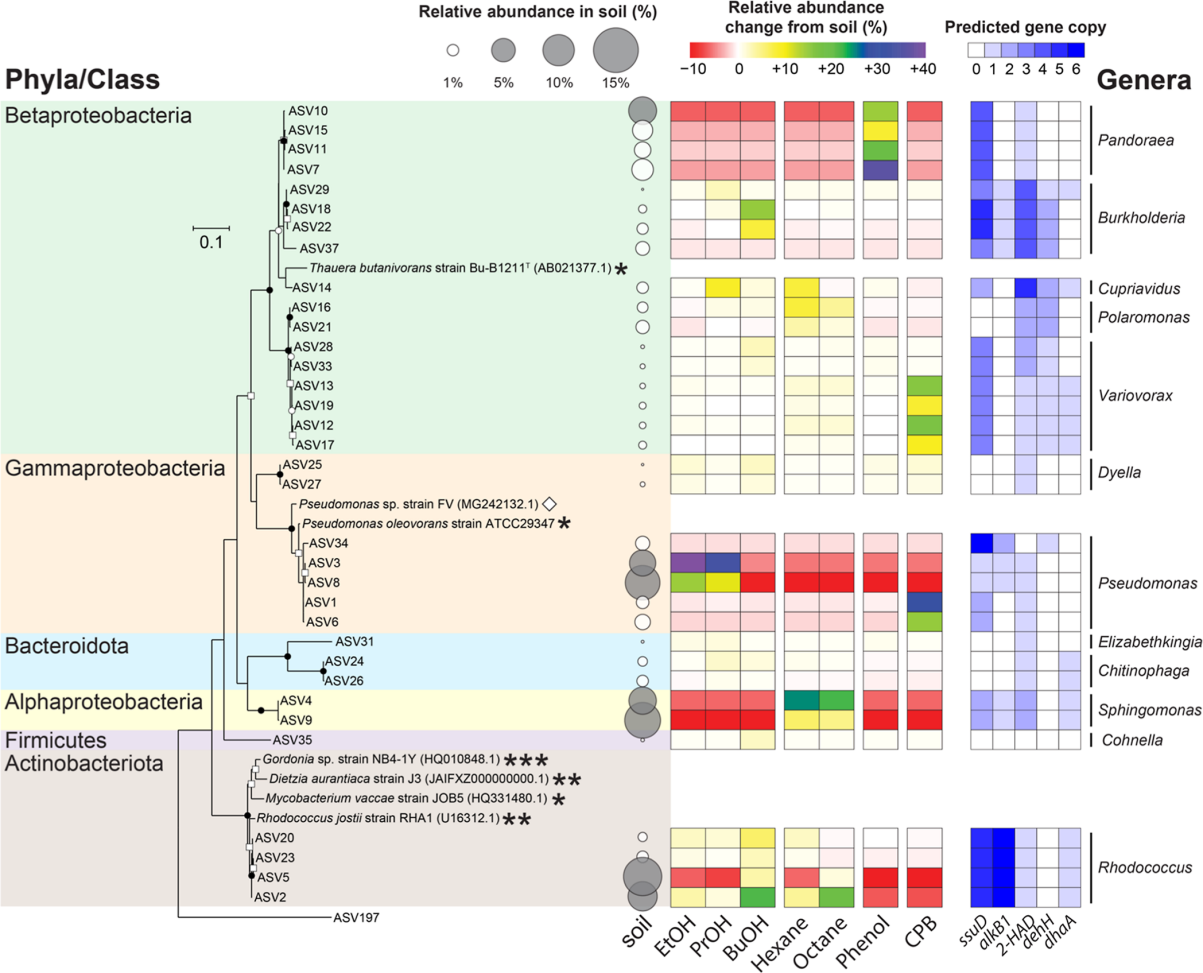
ASV-level microbial community structure of the original soil and respective enrichment cultures. The relative abundance is averaged by triplicate samples. Only 34 ASVs with relative abundance ≥1.0% from at least one sample type are displayed. Shaded bubbles represent the initially abundant ASVs with relative abundance ≥5.0% in the original soil. Heatmap next to the bubble plot indicate changes in relative abundances of respective ASVs in respect to how much was present in the original soil. The right (white-blue) heatmap depicts predicted gene counts pertaining to desulfonation (*ssuD*) and defluorination (*alkB1*, *2-HAD*, *dehH*, and *dhaA*) in respective ASVs. Known PFAS degraders are marked with asterisks (*: 6:2 FtOH degrader, **: 6:2 FtS degrader, and ***: 6:2 FtSaB and 6:2 FtS degrader), and a known CPB degrader is marked with an open diamond (◇). Phylogenetic tree branching points show percentages of clustering sequences by closed circles (>90%), open circles (>70%), and open squares (>50%) based on 1000 replicates of bootstrap analysis. Bar unit indicates 10% nucleotide substitution. An archaeon, ASV197 (Bathyarchaeia), is placed as an outgroup.

Enrichment with different classes of carbon sources resulted in significant shifts in microbial community structure and dominating main populations apart from the original sandy soil in both the phylum- and genus-level (Figure S12). Furthermore, the microbial communities were relatively clustered based on the type of carbon sources, such as alcohols, alkanes, an aromatic compound, and a hydrocarbon surfactant (Figure S13). However, the BuOH-enriched culture was an exception which was apart from EtOH- and PrOH-enriched cultures. Specifically, in the original soil microbiome, the abundant (>5%) amplicon sequence variants (ASVs) were related with *Pseudomonas*, *Rhodococcus*, *Sphingomonas*, and *Pandoraea* (Figure 4). Upon carbon source amendment, EtOH and PrOH significantly promoted the enrichment of *Pseudomonas* (ASV3 and ASV8). On the other hand, BuOH enriched an entirely different community mainly dominated by *Rhodococcus* (ASV5, ASV2, ASV20, and ASV23), *Burkholderia* (ASV18 and ASV22), *Variovorax* (ASV28 and ASV33), and *Dyella* (ASV25 and ASV27). The hexane- and octane-enriched cultures were relatively similar, with an increase in 10–20% *Sphingomonas* (ASV4 and ASV9). However, octane facilitated the more dominant *Rhodococcus* (ASV5 and ASV2). Phenol and CPB allowed the enrichment of only a few unique and dominant populations. For example, phenol predominantly enriched *Pandoraea* (ASV10, ASV15, ASV11, and ASV7) while CPB enriched *Variovorax* (ASV13, ASV19, ASV12, and ASV17) and *Pseudomonas* (ASV1 and ASV6) almost exclusively.

To better understand the desulfonation and defluorination capabilities by respective ASVs, a heatmap shows the predicted gene counts encoding for enzymes known for desulfonation (*ssuD*) of sulfur-containing PFAS such as 6:2 FtS, and for defluorination (*alkB1*, *2-HAD*, *dehH*, and *dhaA*) of 6:2 FtOH (Figure 4).^35,37,38^ Among the predicted genes, *ssuD* and *2-HAD* were frequently predicted and are present in many ASVs that would have the ability to desulfonate and defluorinate precursors.

Further alpha diversity, co-occurrence analysis, and the stochasticity of respective enrichment cultures revealed additional dynamics among the dominant and rare species. In general, the BuOH-enriched culture showed highest diversity, while hexane-, phenol-, and CPB-enriched culture showed low diversity (Table S5). Limited counts of correlations were present in all enrichment cultures among the dominant species except for the EtOH- and PrOH-enriched cultures, which had low PFAS degrading capabilities (Figure S14 and S15). Particularly, positive correlations between *Rhodococcus* and *Dyella* were observed in the BuOH-enriched culture, which had effective 6:2 FtS degradation. *Dyella* has been reported to degrade aromatic compounds such as biphenyl, azo dyes, and triclosan.^71−73^ However, no known PFAS degradation capabilities have been reported and what interaction *Dyella* has with other microbial populations remain unknown. The complex networks for EtOH- and PrOH-enriched cultures centered around *Pseudomonas* (ASV3 and ASV8) could potentially reflect significant competition or exploitation which results in inefficient PFAS degradation.^74,75^ The BuOH- and octane-enriched cultures exhibited the highest stochasticity which might allow extra room for microbial populations to exhibit more dynamic strategies for PFAS degradation through mechanisms such as random dispersion, random birth-death, and community drifts (Figure S16).^74^ However, high stochasticity would not always ensure effective PFAS degradation as the dominance of active PFAS degraders should also be considered. The lack of active degraders might have been the cause for the PrOH-enriched culture to exhibit ineffective PFAS degradation despite high stochasticity. Additional discussion is provided in Supporting Information Section S5.

### 3.4 Unique or Dominant ASVs Have Specific Roles on Precursor Biodegradation

Redundancy analysis (RDA) was performed to probe for correlations among microbes in the enrichment cultures and metabolites from biotransformation of 6:2 FtOH (Figure S17) and 6:2 FtS (Figure S18). In the RDA plots, the angles between all vectors reflect their (linear) correlation. For example, uncorrelation is defined when the angle between vectors = 90°. Positive and negative correlation is defined when the angles are <90 and >90°, respectively. Two genera, *Rhodococcus* and *Burkholderia*, were identified to significantly correlate with the metabolites from 6:2 FtOH (Figure S17) and 6:2 FtS (Figure S18).

While the community structures of EtOH- and PrOH-enriched cultures are similar, *Burkholderia* (ASV29, ASV18, and ASV22), *Cupriavidus* (ASV14), and *Polaromonas* (ASV16 and ASV21) were more dominant in the PrOH-enriched culture. Regardless, in both communities, the dominant *Pseudomonas* (ASV3 and ASV8) could potentially be most likely involved in precursor biodegradation as they were closely related to strains previously known to degrade FtOHs, PAP, and 6:2 FtS.^37,38,40^ The BuOH-enriched culture had dominant *Rhodococcus* (ASV20, ASV23, ASV2, and ASV5) and *Burkholderia* (ASV18 and ASV22), suggesting that they might be important genera pertaining to FT-based PFAS degradation (Figure 4).

*Rhodococcus* was abundant in other enrichments and thus had high niche width values (Table S6), indicating that these ASVs were generalists. These generalists might play an important role in precursor biodegradation (particularly via cometabolic degradation) as they are metabolically diverse and capable of expressing enzymes with a broader substrate range. Consequently, *R. jostii* RHA1, *Gordonia* sp. NB4-1Y, and *Dietzia aurantiaca* J3 can be also considered as metabolically diverse generalists as they are close relatives which could degrade 6:2 FtS or 6:2 FtSaB.^35,36,41,42^ While the roles of *Burkholderia* is unknown, some *Burkholderia* are fluoroacetate degraders^76^ which could also potentially defluorinate FtOHs and 5:3 FtCA.^38^

Similar microbial structures were also observed for alkane-enriched cultures. *Rhodococcus*, *Sphingomonas*, *Cupriavidus*, and *Polaromonas* were found to be associated with the degradation of 6:2 FtOH (Figure S17) and 6:2 FtS (Figure S18). Although higher association toward 6:2 FtOH degradation was observed (Figure S17), no known PFAS degrading mechanisms of *Sphingomonas* have been reported. However, the genus have been frequently reported as speculative players in 8:2 FtOH^61^ and 6:2 FtS^66^ degradation. Interestingly, there was less *Cupriavidus* (ASV14) but more *Rhodococcus* (ASV2) in the octane-grown culture than that in the hexane-grown culture. The subtle difference in the microbial composition could have allowed the octane-enriched culture to improve 6:2 FtS degradation. The microbial community structures of phenol- and CPB-enriched cultures were significantly less diverse. For example, only ASVs associated with *Pseudomonas* and *Pandoraea* in the CPB-enriched culture and *Variovorax* in the phenol-enriched culture were significantly dominant. These genera have been known to degrade various aromatic xenobiotics^77−79^ and CPB.^48^ However, only *Pseudomonas* has been characterized of being capable of degrading PFAS.^37^*Variovorax* has no reports of known PFAS degradation capabilities but potentially possess diverse genes pertaining to PFAS degradation.^80^ Further studies are required to identify their roles and interactions from PFAS degradation scenarios in AFFF-impacted sites.

From the dual-culture degradation experiments, only the combination of phenol- and CPB-enriched cultures resulted in a lower 6:2 FtOH defluorination (Figure S2B). Presumably, *Pandoraea* might not be compatible with *Variovorax* or *Pseudomonas* for a 6:2 FtOH defluorination. The potential loss of the defluorinating capability of the mixed CPB- and phenol-enriched cultures could also be described by the predictive gene profiles which is described in the next section. Overall, the increased defluorination of other remaining dual enrichment culture combinations results suggested synergistic effects of FtOH degradation by the microbes (Figure S3).

### 3.5 Predictive Functional Genes Profiles Are Linked to the Precursor

**Figure 5:**
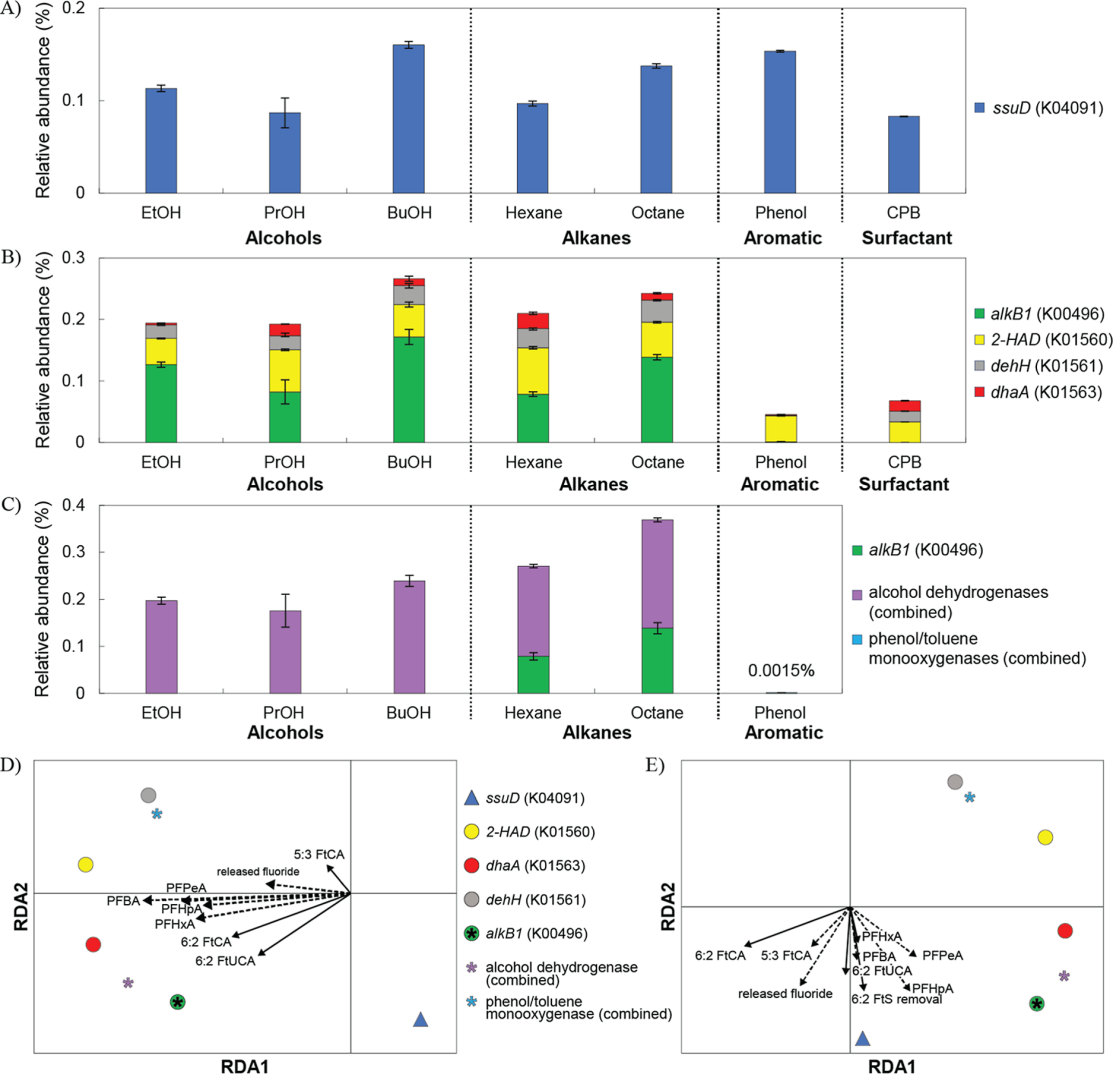
Relative abundances of predicted functional genes responsible for (A) PFAS desulfonation, (B) PFAS defluorination, and (C) respective carbon source metabolism. Bars represent ranges of duplicate sample predictions. Catabolic genes for CPB degradation were not predicted as inducible CPB metabolic genes are unknown. RDA of (D) 6:2 FtOH and (E) 6:2 FtS degradation pertaining variables correlated with predicted gene relative abundance patterns (triangle: PFAS desulfonation; circle: PFAS defluorination; and asterisk: carbon source metabolism). Bold arrows within the RDA indicate variables with significance (*p* < 0.05), while dashed arrows are less significant (*p* > 0.05). The released fluoride variable for the 6:2 FtS RDA was input from the 20 mg/L 6:2 FtS degradation experiment.

Functional genes encoding enzymes for desulfonation (Figure 5A) and defluorination (Figure 5B),^35,41,80,81^ and carbon source metabolisms (Figure 5C)^37,82−85^ were predicted. For desulfonation, *ssu*D (K04091) encoding alkanesulfonate monooxygenase was predicted. Previous studies identified *ssuD*, expressed under S-limited conditions,^62,76^ as a crucial gene to initiate 6:2 FtS and 6:2 FtSaB desulfonation.^35^ Accordingly, the predicted *ssuD* gene was abundant in the BuOH-, octane-, phenol-, and CPB-enriched cultures which correspondingly had the highest 6:2 FtS removal (Figure 5A).

For defluorination enzymes reported from literature, *dehH* (K01561) encoding haloacetate dehalogenase, *dhaA* (K01563) encoding haloalkane dehalogenase, and *2-HAD* (K01560) encoding for 2-haloacid dehalogenase, *alkB1* (K00496) encoding alkane 1-monooxygenase which could participate in both defluorination and metabolic alkane degradation^37^ were also predicted (Figure 5B). The *alkB1* within *Rhodococcus* is identified as an important gene for the defluorination of 6:2 FtS after desulfonation.^35,36^ The *alkB1* is also expressed for octane metabolism along with 6:2 FtOH defluorination.^37^ The high abundance of *alkB1* in BuOH- and octane-enriched cultures could have resulted in effective FtOH and 6:2 FtS degradation that requires defluorination. The profile of other defluorinating genes was highly dominant in the BuOH-, hexane-, and octane-enriched cultures, which correspondingly resulted in effective FtOH degradation observed in Figures 1 and S2–S4. Significant lack of the defluorinating genes might have been the cause of the decreased 6:2 FtOH defluorination by the combined phenol- and CPB-enriched cultures.

Enzymes involved in the oxidation and utilization of different classes of carbon sources were also predicted (Figure 5C). A group of alcohol hydrogenases (K00001, K00114, K13953, K13954, K18369, and K08325) were predicted (Figure S19A). The high abundance of alcohol hydrogenases predicted in the alcohol- and alkane-enriched cultures was not surprising as alcohols are the immediate products of aerobic alkane oxidation. Alcohols such as PrOH and BuOH are also known to induce propane and butane monooxygenases, respectively.^86^ In fact, alcohols have been frequently used and resulted in effective PFAS removal in biodegradation studies.^35^ For alkane oxidation, again, *alkB1* was predicted. For phenol-enriched cultures, *tmoA* (K15760), *dmpL* (K16243), *dmpN* (K16242), *dmpO* (K16245), and a phenol 2-monooxygenase (K03380) encoding the oxygenase components for toluene or phenol oxidation were predicted (Figure S19B). However, no metabolic genes responsible for CPB degradation were predicted.^74^ As CPB is present in the AFFF formula, more study is required to link the roles and enzymes of CPB-degrading cultures responsible for PFAS precursor degradation.

Through RDA, all of the predicted defluorination genes described above showed positive correlations with 6:2 FtOH biodegradation metabolites (Figure 5D). Specifically, *alkB1* is positively associated with the presence of upper pathway metabolites (from 6:2 FtOH to 6:2 FtUCA and 6:2 FtCA) while *dehH* is positively associated with 5:3 FtCA. This observation was supported by previous findings that a fluoroacetate dehalogenase-expressing *P. fluorescens* not only produced 5:3 FtCA from 6:2 FtOH biodegradation but also degraded 5:3 FtCA to 4:3 FtCA.^38^ Also, the *dehH* from *Delftia acidovorans* have shown to defluorinate PFAS,^87^ and from *R. jostii* RHA1 have shown to defluorinate 4:2 FtOH.^35^ High correlation of phenol/toluene monooxygenase with FtOH biodegradation were supported by this study and by a previous observation of enhanced 6:2 FtTAoS biodegradation by amending BTEX.^45^ Yet, future studies are needed to directly examine the role of phenol/toluene monooxygenases on precursor degradation.

The significance of the *ssuD* was observed through being strongly associated with the 6:2 FtS degradation variables (Figure 5E). This finding was strongly supported by previous and current studies in which desulfonation is the rate-limiting step for defluorination of sulfur-containing PFAS precursors. Overall, using RDA analysis, strong correlations of genes encoding enzymes involving desulfonation, defluorination, and carbon source metabolisms are clearly delineated with respect to types of FT-based precursors containing with or without sulfur.

## 4 Environmental Implications

This study reports the first systematic evaluation of the effects of different classes of carbon sources, including a surfactant, CPB, on shaping the soil microbiome favorable for PFAS degradation. In response to the different carbon sources, rare species in the original soil microbiome become abundant, and their unique catabolic function was thus unlocked, leading to different FT-based PFAS precursor degradation abilities. Except for 4:2 FtOH, the dependency of the defluorination of 6:2 FtOH and 8:2 FtOH on the increasing alcohol chain length was noted. Also, combinations of dual enrichment cultures enhanced defluorination extents and increased fluoride release from 6:2 FtOH, suggesting a new biostimulation strategy of using multiclass carbon sources to promote precursor biodegradation. Depending on the types of precursors and the available carbon sources present at the contaminated sites, the presence of different classes of carbon sources (contaminated with BTEX, organic solvents, or other hydrocarbon surfactants) can be used to anticipate how PFAS biotransformation would occur based on the acclimated microbial populations. This aspect can be particularly important for sites containing both PFAS and other cocontaminants.

Even within the same classes, BuOH is more effective than EtOH and PrOH in PFAS degradation. Octane is more effective than hexane. Dominant ASVs associated with *Burkholderia* and *Rhodococcus* were close relatives to known FtOHs and 6:2 FtS degraders. Additionally, despite low diversities within the phenol- and CPB-enriched cultures, FT-based PFAS biodegradation occurred. The occurrence suggests that *Pandoraea* and *Variovorax* in the phenol- and CPB-enriched communities, respectively, might harbor enzymes and/or catabolic genes capable of degrading precursors. Yet, little is known about the microbes within these two genera, which warrants further investigation.

Suitable biomarkers that can confirm, monitor, and assess the treatment effectiveness of PFAS precursors can be a valuable tool for managing PFAS-impacted sites. Many studies have attempted to use dominant species and occurrence frequency in PFAS-exposed communities to draw association with their degradation ability. However, in this study, we found that such an approach might not be valid and presents significant drawbacks. For example, *Pseudomonas* was dominant in EtOH-, PrOH-, and CPB-enriched communities but was much less in the BuOH-enriched community. However, very poor 6:2 FtS degradation by EtOH- and PrOH-enriched cultures, medium 6:2 FtS removal by CPB-enriched cultures, but a near complete removal of 6:2 FtS by BuOH-enriched cultures were observed. These results highlighted the limitations of such an approach to identify microbes as suitable biomarkers. In fact, through RDA analysis, we have demonstrated that using ASVs and degradation metabolites of 6:2 FtOH and 6:2 FtS, one can observe positive or negative correlations among the carbon source types, ASVs in the enriched communities, and metabolites of the precursors. Accordingly, key PFAS degraders can be further identified and potentially developed as suitable biomarkers.

Amplicon-based functional prediction, such as using 16S rRNA gene sequences to identify assumptive functional genes, has two main limitations: (i) predictions are limited by the reference genomes in the existing database, leading to rare, environment-specific functions harder to detect and (ii) unable to provide sufficient resolution to distinguish strain-specific functionality.^88^ In this study, dominant microbial populations (i.e., ASV) were not directly linked to PFAS biodegradation. However, by utilizing a phylogenetic tree, as described in this study, it is relatively easy and possible to identify which microbial populations within the community (i.e., ASV) were closely related to known PFAS degraders (Figure 4). As some functional genes might be commonly shared among different taxa, identifying genes involved in PFAS degradation would be more useful and practical. Using RDA along with the predicted functional genes and detected degradation metabolites, this study was able to highlight the important roles of the desulfonating (*ssuD*) and defluorinating (*2-HAD*, *dehH*, *dhaA*, and *alkB1*) genes in 6:2 FtS and 6:2 FtOH degradation. The results suggested that these genes are promising candidates to serve as biomarkers. For sulfur-containing PFAS precursors like 6:2 FtS, cooperation between different classes of genes responsible for desulfonation and defluorination would be important as the desulfonation step is a rate-limiting step for 6:2 FtS degradation. Future work is needed to isolate pure strains from these enrichments for a detailed study of PFAS degradation, gene identification, and expression during the degradation.

As metabolic genes for CPB degradation are currently unknown, future studies to investigate CPB-degrading strains and the functional genes that might be involved in precursor biodegradation are warranted. Additionally, enzymatic studies to confirm the ability of phenol/toluene monooxygenase to inhibit PFAS degradation are also needed.

## Supporting Information Available

Details regarding chemicals, soil enrichment process, SBR operation, sample collection and experimental setup, 16r RNA gene sequencing and amplicon analysis; experimental results and discussions; target PFAS list, soil properties and composition; AMS and S-free AMS media composition, alpha diversity, and niche width; SBR operation; PFAS degradation metabolites and defluorination results; suspect-screening results; PFAS degradation pathway; phyla/class- and genus-level microbial community structure; principle coordinate analysis plot; co-occurrence analysis; stochasticity; RDA; and carbon source metabolic gene profile (PDF)
