# Supplementary material for: Nexus of Soil Microbiomes, Genes, Classes of Carbon Substrates, and Biotransformation of Fluorotelomer-Based Precursors
Source: Environ Sci Technol. 2024 Nov 6;58(46):20553–65. doi: 10.1021/acs.est.4c06471 (PMC11580179; doi:10.1021/acs.est.4c06471)
Supplement: Supplementary file 1 — es4c06471_si_001.pdf [file es4c06471_si_001.pdf]

Supporting Information

for

The Nexus of Soil Microbiomes, Genes, Classes of Carbon Substrates, and Biotransformation of Fluorotelomer-Based Precursors

Jinha Kim[1], Scott W. Leonard[2], Mariann Inga Van Meter[3],

Mitchell L. Kim-Fu[3], Dunping Cao[3], Jennifer A. Field[2], Kung-Hui Chu[1,*]

[1]Zachry Department of Civil and Environmental Engineering, Texas A&M University, College Station, TX, 77843, USA

[2]Department of Environmental and Molecular Toxicology, Oregon State University, Corvallis, OR, 97331, USA

[3]Department of Chemistry, Oregon State University, Corvallis, OR, 97331, USA

*Corresponding author:

Kung-Hui Chu, Zachry Department of Civil and Environmental Engineering
3136 TAMU, Texas A&M University, College Station, TX 77843, USA
Phone: 979-845-1403, Fax: 979-862-1542
Email: kchu@civil.tamu.edu

This supporting information includes 42 pages of text describing Sections S1-S5, Tables S1-S6, Figures S1-S19, and references.

Table of Content

Section S1. Materials and Methods……………………………………………………………S6

a. Chemicals………………………………………………………………………………..S6

b. Soil enrichment and sequencing batch reactor operation……………………………...S6

c. Experimental setup for sulfur-containing PFAS degradation……………………………S6

d. Target- and suspect-screening LC-QToF operation methods……………………………S7

e. Microbial community analysis………………………………………………………..S7

Section S2. FtOH Biodegradation by Mono-enrichment Cultures………………………S9

Section S3. 6:2 FtOH Biodegradation by Dual-enrichment Cultures……………………S10

Section S4. 6:2 FtSaB Biodegradation by Enrichment Cultures…………………………S11

Section S5. Alpha Diversity, Co-occurrence Analysis, and Community Assemblage Mechanism……………………………………………………………………S12

List of Tables

Table S1. Target PFAS (negative mode), acronym, neutral molecular formula, and surrogate standard for analysis by QToF…………………………………………………S13

Table S2. Target PFAS (positive mode), acronym, neutral molecular formula, and surrogate standard for analysis by QToF……………………………………………………..S15

Table S3. Properties and compositions of the sandy soil used in this study………………S16

Table S4. Ammonia mineral salts (AMS) media and modified S-free AMS media composition and concentrations. Prepared media was adjusted to pH 7 with HCl or NaOH. Footnotes indicate trace SO_4^{2-} residuals present in the utilized chemical stocks based on provided assays by vendor. Specific assay is unknown for chemicals without a footnote……………………………………………………………S17

Table S5. Alpha diversity indices of original sandy soil (inoculum) and respective microbial enrichment communities. The indices depict averages and standard deviations from random 10-time sampling of respective triplicate samples at sequence depth 33,000……………………………………………………………………………..S18

Table S6. Niche width (i.e., Levins' niche breadth) of core 34 ASVs. ASVs with high niche width values are considered generalists while specialists have low niche width values……………………………………………………………………………...S19

List of Figures

Figure S1. Time course biomass (as optical density at OD_{600}) and CO_2 concentration in sequencing batch reactors receiving difference carbon sources: A) EtOH, B) PrOH, C) BuOH, D) Hexane, E) Octane, F) Phenol, or G) CPB……………………S20

Figure S2. Degradation metabolites and fluoride levels detected from A) 4:2 FtOH, B) 6:2 FtOH, and C) 8:2 FtOH resting cell degradation experiments after three days. Fluoride equivalent (F eq) was used for all metabolites. For example, 1 mole of 3:3 FtCA = 7 moles of F eq; 1 mole of PFBA = 7 moles of F eq; 1 mole of 5:3 FtCA = 11 moles of F eq; and 1 mole of PFHxA = 11 moles of F eq. The right graph shows zoomed-in profile of measured released fluoride and FtOH degradation metabolites excluding FtCAs and FtUCAs. Bars represent ranges of duplicate sample measurements……………………………………………………S22

Figure S3. Fluoride released from 6:2 FtOH biodegradation by dual enrichment cultures are shown in blue. The experiments were conducted using 1:1 (vol/vol) of dual enrichments. Assuming there are no synergistic effects on the defluorination of 6:2 FtOH, the expected fluoride release by the dual cultures is 50% of the summation of fluoride release by the two individual cultures as measured in Figure 1. Bars represent ranges of duplicate sample measurements……………………………S23

Figure S4. Degradation metabolites and fluoride detected from the mono- and dual-culture resting cell 6:2 FtOH degradation experiments. The right graph shows zoomed-in profile of 6:2 FtOH degradation metabolites of only PFHpA, PFHxA, PFPeA, PFBA, 5:3 FtCA, and the measured released fluoride. Bars represent ranges of duplicate sample measurements…………………………………………...S24

Figure S5. Total fluoride mass detected from 6:2 FtS growth experiments after eight days. The initial 6:2 FtS concentration was 20 mg/L (46.7 μmol/L = 607.1 μmole/L F eq.). Bars represent ranges of duplicate sample measurements……………………S25

Figure S6. PFAS concentrations of the no-cell controls on Day 0 and the killed-cell controls on Day 7 and Day 9 from A) positive mode and B) negative mode target-screening methods of the 6:2 FtSaB degradation experiment samples. Bars represent ranges of duplicate sample measurements.…………………………………………S26

Figure S7. Concentrations of 6:2 FtSaB in killed-cell controls and samples from the 6:2 FtSaB degradation experiments on Day 7 and Day 9 by A) BuOH-, B) octane-, C) phenol-, and D) CPB-enriched cultures. Positive mode target-screening method was used. Bars represent ranges of duplicate sample measurements……………………S27

Figure S8. Suspect analysis of the no-cell controls on Day 0 and the killed-cell controls on Day 7 and Day 9 and samples of the 6:2 FtSaB degradation experiments. By using suspect-screening methods, three suspects were identified from samples. They are A) NIST suspect list #3736 (structure of 6:2 FtSaB) and B) NIST suspect list #881

(structure of 6:2 FtSaAm; tertiary amine) and #3825 (structure of 6:2 FtSaAm; secondary amine). Asterisk (*) indicates only a single signal measurement from duplicate samples. Bars represent ranges of duplicate sample measurements…..S28

Figure S9. Comparison of NIST suspect list #3736 (structure of 6:2 FtSaB) signal areas from killed-cell controls and those from the samples of the 6:2 FtSaB degradation experiments. Samples are from A) BuOH-, B) octane-, C) phenol-, and D) CPB-enriched cultures on Day 7 and Day 9. Bars represent ranges of duplicate sample measurements……………………………………………………………………..S29

Figure S10. NIST suspect list #881 (structure of 6:2 FtSaAm; tertiary amine) and #3825 (structure of 6:2 FtSaAm; secondary amine) signal areas from killed-cell controls and samples of the 6:2 FtSaB degradation experiments. Samples are from A) BuOH-, B) octane-, C) phenol-, and D) CPB-enriched cultures on Day 7 and Day 9. Asterisk (*) indicates only a single signal measurement from duplicate samples. Bars represent ranges of duplicate sample measurements………………………S30

Figure S11. Proposed 6:2 FtSaB or 6:2 FtS degradation pathway adapted with references.[1-4] Compounds in blue and yellow background have been identified through target-screening and suspect-screening methods in this study, respectively from all the 6:2 FtSaB, 6:2 FtS, and 6:2 FtOH degradation experiments. Black dashed arrows indicate uncertain reactions. Green and red dashed arrows indicate the steps in which desulfonation and defluorination occur. However, to our best knowledge, defluorination genes involved in the defluorination steps of the degradation pathway remain unknown…………………………………………………....S31

Figure S12. A) Phylum/class- and B) genus-level microbial community structure of original soil and respective enrichment cultures. The relative abundance is averaged from triplicate samples……………………………………………………….......S32

Figure S13. Principle coordinate analysis (PCoA) plot on the weighted UniFrac distance matrix referring beta-diversity of original soil and respective enrichment communities at sequence depth 33,000……………………………………………………....S33

Figure S14. Co-occurrence network analysis from triplicate samples of alcohol (EtOH, PrOH, and BuOH) enrichment cultures. Only ASVs that has relative abundance >0% within all triplicate samples were considered for analysis. Nodes are highlighted in green, light green, yellow for relative abundances being >15%, >5%, and >1%, respectively. Pearson correlation coefficient values with p-value <0.05 were used for the display of edges in which green represents positive (≥0.5) and red represents negative correlations (≤-0.5)…………………………………………………S34

Figure S15. Co-occurrence network analysis from triplicate samples of alkane (hexane and octane), phenol, and CPB enrichment cultures. Only ASVs that has relative abundance >0% within all triplicate samples were considered for analysis. Nodes are highlighted in green, light green, yellow for relative abundances being >15%,

>5%, and >1%, respectively. Pearson correlation coefficient values with p-value <0.05 were used for the display of edges in which green represents positive (≥0.5) and red represents negative correlations (≤-0.5)……………………………...S35

Figure S16. Box and whisker plot (○ = mean; × = outlier) of the relative contribution of the stochastic process in the microbial community assemblage. Stochasticity is represented as the estimated taxonomic normalized stochasticity ratio (tNST) index calculated from A) Jaccard (unweighted presence/absence of ASVs) and B) Bray-Curtis (relative abundance weighted presence/absence of ASVs) distance matrices by bootstrapping 1,000 times of triplicate samples. 1-tNST value represents the relative contribution of the deterministic process………………………………S36

Figure S17. A) Redundancy analysis (RDA) depicting correlation between 6:2 FtOH degradation variables and ASV-level microbial composition. The gray area is zoomed-in to show B) correlation between 6:2 FtOH degradation variables and associated ASVs. Pie chart reflects the presence of the ASV within a designated enrichment culture (i.e., purple = EtOH, brown = PrOH, orange = BuOH, blue = hexane, red = octane, pink = phenol, green = CPB). Circles are large (genera are also bolded with an asterisk) if the relative abundance of the corresponding ASV is >5% within the designated enrichment culture. Circles are small and dashed if the ASV is <5% and >1%. Bold arrows within the RDA indicate variables with significance (p<0.05) while dashed arrows are less significant (p>0.05)……….S37

Figure S18. A) Redundancy analysis (RDA) depicting correlation between 6:2 FtS degradation variables and ASV-level microbial composition. The gray area is zoomed-in to show B) correlation between 6:2 FtS degradation variables and associated ASVs. Pie chart reflects the presence of the ASV within a designated enrichment culture (i.e., purple = EtOH, brown = PrOH, orange = BuOH, blue = hexane, red = octane, pink = phenol, green = CPB). Circles are large (genera are also bolded with an asterisk) if the relative abundance of the corresponding ASV is >5% within the designated enrichment culture. Circles are small and dashed if the ASV is <5% and >1%. Bold arrows within the RDA indicate variables with significance (p<0.05) while dashed arrows are less significant (p>0.05). The released fluoride variable for the 6:2 FtS RDA was input from the 20 mg/L 6:2 FtS degradation experiment……………………………………………………………...S38

Figure S19. Relative abundance of predicted genes pertaining to A) alcohol and B) phenol/toluene oxidation………..…………………………………………....S39

Section S1. Materials and Methods

a. Chemicals

Ethanol (EtOH; 100%; CAS # 64-17-5) was purchased from Koptec (Decon Labs, PA). 1-Propanol (PrOH; ≥99.5%; CAS # 71-23-8) and the graphitized non-porous carbon powder Supelclean™ ENVI-Carb™ (100%; CAS # 7782-42-5) were purchased from Sigma-Aldrich (St. Louis, MO). 1-Butanol (BuOH; ≥99.4%; CAS # 71-36-3) and hexane (≥95% [ACS grade]; CAS # 110-54-3) were purchased from Fisher Scientific (Pittsburgh, PA). Phenol (100%; CAS # 108-95-2) was purchased from J. T. Baker (Phillipsburg, NJ). Octane (97%; CAS # 111-65-9) and acetonitrile (99%; CAS # 75-05-8) were purchased from Acros Organics (Morris Plains, NJ). Cocamidopropyl betaine (CPB; 95%; CAS # 61789-40-0) was purchased from Combi-Blocks (San Diego, CA).

b. Soil enrichment and sequencing batch reactor operation

Soil enrichment cultures were initiated in a series of 20-mL glass vials containing using 0.2 g of the soil in 2 mL ammonia mineral salt (AMS) medium.[5, 6] Each vial was then spiked with one of the following carbon sources at an initial concentration of 0.05 % EtOH, PrOH, BuOH, hexane, octane, phenol, or CPB. The vials were incubated at room temperature with shaking at 160 rpm. Once the enrichment cultures showed signs of growth (i.e., increased turbidity, CO_2 production, and O_2 consumption), the enrichments were subcultured to new vials containing fresh AMS medium and their respective carbon sources. After more than five subcultures, the enrichment cultures were used as seeds to establish quasi-steady-state enrichment cultures in a series of 500-mL flasks at a 50-mL working volume. Respective carbon sources were amended, and the flasks were operated as sequencing batch reactors (SBR) at room temperature.

Each flask was inoculated with a seed culture at an optical density (OD_{600}) of 0.05 and its respective carbon source in a working volume of 50 mL AMS media. The inoculated flasks were then operated while mixing at 160 rpm on a shaker. To establish quasi-steady state enrichment cultures for experimental use, solid retention time (SRT) of 1 day was used to establish EtOH-, PrOH-, BuOH-, phenol- and CPB-enriched cultures. For hexane- and octane-enriched cultures, a 2-day SRT was used. The stable enrichment cultures in the SBRs were established based on the stable measurements of OD_{600} and CO_2 production observed over more than three times of the SRTs. The EtOH- and BuOH-enriched cultures achieved steady biomass concentrations at OD_{600} = ~0.8, while PrOH-, hexane-, octane-, phenol- and CPB-enriched cultures achieved steady biomass concentrations at OD_{600} = 0.5~0.6 (Figure S1). Except for the phenol-enriched culture which achieved ~4% (v/v) CO_2, all other enrichment cultures achieved ~1% (v/v) CO_2 production in the SBR headspaces. The stable cultures were collected for microbial community analysis and used for PFAS precursor biodegradation tests.

c. Experimental setup for sulfur-containing PFAS degradation

The 6:2 FtSaB growth experiments were conducted similarly in 20-mL HDPE vials as described for the 6:2 FtS growth experiments. The HDPE vials were sealed with silicone septa and spiked with 4 µL of the 6:2 FtSaB stock solution (1 g/L in 10% MeOH) to bring an initial 6:2 FtSaB concentration of 4 mg/L. In these experiments, only BuOH-, octane-, phenol-, and CPB-enriched cultures were used as inocula. The vials had two groups. Group 1 vials were spiked with

carbon sources only on Day 0. Group 2 vials had additional spikes of carbon sources. For example, 0.05% BuOH, phenol, and CPB were additionally spiked on Days 3 and 7. Octane was only spiked again on Day 5. Group 1 and 2 vials were incubated for seven and nine days, respectively, at room temperature before being sacrificed for PFAS analysis. Positive controls contained only enrichment cultures with their respective carbon sources in AMS medium. The growth of cultures in the positive controls were monitored based on increase of optical densities and oxygen consumption. Two types of negative controls, killed-cell control (i.e., cell autoclaved) and only PFAS in growth medium control, were also set. All experiments were conducted in duplicate.

d. Target- and suspect-screening LC-QToF operation methods

A total of 50 target PFAS found in negative mode (Table S1) and 26 stable-isotope labelled standards and five target PFAS found in positive mode (Table S2) and 1 stable-isotope labelled standard were employed for quantitative analysis (Wellington Laboratories, Guelph, ON, Canada), except d3-Prometon which was purchased from CDN (Pointe-Claire, QC H9R 1H1, Canada).

Samples received at OSU were spiked with 26 stable isotope-labelled (surrogate) standards found in negative mode and 1 stable isotope-labelled (surrogate) standard for positive mode PFAS and analyzed for target and suspect PFAS. If any PFAS were off scale, the sample was diluted below the detection of spiked surrogate, respiked and analyzed.

Analyses for target and suspect PFAS were performed by liquid chromatography quadrupole time of flight mass spectrometry (LC-QToF) on a SCIEX 7600 (Framingham, MA) under negative- and positive-mode ionization with SWATH® Data-Independent Acquisition. Precursor ion data were collected for m/z 100-1200 Da with an accumulation time = 200 ms. The ion spray voltage was set at –4500 V (ESI-) and temperature was set to 525°C with an ion source gas pressure of 60 psi, a curtain gas pressure of 35 psi, and a collision (CAD) gas pressure of 10 psi. For QToF scanning, the collision energy was set to -5 V and the declustering potential was set to -20 V, both with a spread of 0 V. Product ion (MS/MS) scanning for m/z 50-1200 Da was conducted in the following mass m/z windows (Da): 100 − 150, 149 − 200, 199 − 250, 249 − 300, 349 − 400, 399 − 450, 449 − 550, 549 – 650, 649 – 800, 799 – 1200. For each SWATH window, the accumulation was 50 ms and collision energy was –35 V with spread of 30 V.

Calibration curves were established for the target PFAS found in negative mode (Table S1) and positive mode (Table S2). Calibration standards ranged from 100 to 30,000 (negative-mode PFAS; Table S1) and from 200 to 100,000 ng/L for positive-mode PFAS (Table S2). Calibration check standards were required to be ±30% of nominal values. A third-party reference standard (AccuStandard, New Haven, CT) was used to verify the negative-mode PFAS calibration curve accuracy.

For target PFAS, internal standard quantification was performed and for suspect screening, SciexOS processing software, a custom PFAS library, and the NIST PFAS List (https://data.nist.gov/od/id/mds2-2387)[7] were used to identify suspect PFAS.[8]

e. Microbial community analysis

Briefly, the genomic DNA of the samples was extracted using the FastDNA spin kit for soil (MP Biomedicals, Irvine, CA). Samples were further processed by the Institute for Advanced Materials & Manufacturing Genomics Core at the University of Tennessee, Knoxville. Briefly,

library preparation was conducted by targeting the V3-V4 region and using the Nextera XT library preparation kit (Illumina, San Diego, CA). The prepared library was sequenced in the MiSeq platform (Illumina, San Diego, CA) using a paired-end (2x300 bp) read strategy with the reagent kit v3 (Illumina, San Diego, CA).[9] The obtained raw-sequence reads were processed using qiime2-2022.8.[10] The raw-sequence reads were quality filtered, denoised, merged, and chimera removed using the dada2 plug-in, which generated high-resolution amplicon sequence variants (ASV).[11] Taxa were assigned from the SILVA 138 database, consisting of non-redundant reference sequences clustered by 99% similarity, using BLAST+.[12, 13] The 16S rRNA gene V3-V4 regions of known fluorotelomer-based PFAS or CPB degraders obtained from the National Center for Biotechnology Information (NCBI) and the ATCC genome portal[14] were identified with the primer set (i.e., forward primer: 5'-CCTACGGGNGGCWGCAG-3' and reverse primer: 5'-GACTACHVGGGTATCTAATCC-3') previously used for the library preparation. All sequences were aligned using MAFFT.[15] The phylogenetic tree was construction under MEGA11[16] with the Neighbor-Joining[17] and Maximum Composite Likelihood methods.[18] The Levins' niche width (i.e., niche breadth) and the taxonomic normalized stochasticity indices (tNST) were respectively calculated through the SPAA v0.2.2[19] (https://CRAN.R-project.org/package=spaa) and NST v3.1.10[20] (https://CRAN.R-project.org/package=NST) packages in R. Pearson correlation coefficients were calculated using R and used for co-occurrence network construction assisted with Cytoscape v3.9.1.[21] Functional gene prediction based on the 16S rRNA gene sequence data was conducted through PICRUSt2 v2.4.1.[22] The functional genes were assigned with KEGG Orthology (KO) numbers based on the Kyoto Encyclopedia of Genes and Genomes (KEGG) (http://www.kegg.jp).[23] Redundancy analysis (RDA) was conducted through the vegan v2.6.4 (https://CRAN.R-project.org/package=vegan) package in R.[24]

Raw sequence reads were submitted into NCBI sequence read archive (SRA) under BioProject accession number PRJNA949259.

Section S2. FtOH Biodegradation by Mono-enrichment Cultures

In 4:2 FtOH degradation, only two metabolites, 3:3 FtCA and perfluorobutanoic acid (PFBA), were detected (Figure S2A), while more diverse 6:2 FtOH and 8:2 FtOH degradation metabolites were observed in all enrichment cultures (Figures S2B-S2C). In 6:2 FtOH degradation, trace levels of perfluoroheptanoic acid (PFHpA) were detected only in PrOH-, hexane-, and octane-enriched cultures, (Figure S2B). High amounts of 6:2 FtCA and 6:2 FtUCA were detected in BuOH-, hexane-, and octane-enriched cultures. The levels of 5:3 FtCA were also relatively high in the same three carbon source enrichment conditions and in CPB-enriched culture. Production of 5:2 FtCA from 6:2 FtOH degradation had been previously reported by octane-grown pure strains.[1, 25, 26] This the first observation of 5:3 FtCA production by CPB-enriched cultures. Except by the phenol-enriched cultures, PFBA was detected in all other cultures (Figure S2B). Slightly higher fluoride concentrations from 6:2 FtOH (C6) degradation by the hexane (C6)-enriched cultures and from 8:2 FtOH (C8) degradation by the octane (C8)-enriched cultures were observed. The phenol-enriched cultures showed relatively similar released fluoride (15~20 μM-F) from the degradation of three FtOHs. For the CPB-enriched culture, higher 4:2 FtOH and 6:2 FtOH defluorination were observed compared to 8:2 FtOH defluorination. The trend of higher fluoride release from 6:2 FtOH than 8:2 FtOH defluorination was observed for all the enrichments. However, this trend was not observed for 4:2 FtOH defluorination.

Various FtOH degradation metabolites were detected in the enrichment cultures (Figure S2). Although no measurements of remaining FtOHs were made, the extents of FtOH defluorination can be defined as the summation of fluoride equivalent (F eq) of each of the FtOH metabolites and released fluoride. The fluoride equivalent from the FtOH metabolite portion depicts the fluorides that has the potential to be further released upon more active states of the microorganisms. Unfortunately, a resting cell experiment disallows high activity for further defluorination as no carbon source is supplied for prolonged and continuous activity. Using this defluorination extent approach, PrOH-enriched culture showed the highest extent of 4:2 FtOH defluorination, while octane-enriched culture showed the highest defluorination extent for 6:2 FtOH and 8:2 FtOH.

In 8:2 FtOH degradation, trace levels of perfluorononanoic acid (PFNA) were observed in all the enrichment cultures (Figure S2C). Similarly to 4:2 FtOH and 6:2 FtOH degradation, the major metabolites from 8:2 FtOH biotransformation were 8:2 FtCA and 8:2 FtUCA in the BuOH-, hexane-, and octane-enriched cultures, with less total amount of these two metabolites accumulated in the hexane-enriched cultures (Figure S2C). The trace accumulation of 7:3 FtCA from 8:2 FtOH degradation were also observed in BuOH-, hexane-, octane- and CPB-enriched cultures.

Section S3. 6:2 FtOH Biodegradation by Dual-enrichment Cultures

Resting cells have been used to co-metabolically degrade various types of halogenated organic contaminants[1, 6, 25, 27, 28] by exploiting the use of pre-induced enzymes with the absence of growth or reducing energy providing substrates. Combining various resting cells of pure isolates such as *Pseudomonas butanovora* and *Pseudomonas fluorescens* has been reported to synergistically facilitate 6:2 FtOH biodegradation.[29] Furthermore, bioaugmentation of the same strains within activated sludge could effectively degraded 6:2 FtOH in resting cell conditions without any competing co-substrates. Assuming that there was no synergism on 6:2 FtOH biodegradation between the two individual enrichment cultures, the expected amount of fluoride released from 6:2 FtOH biodegradation is predicted by simply adding the half amounts of fluoride released by two individual enrichment cultures as shown in Figure 1. To determine any synergistic effects of different resting cells on 6:2 FtOH biodegradation, a set of 6:2 FtOH degradation experiments using different combinations of two enrichment cultures (with 1:1 [vol/vol] ratio) were conducted. Enrichment cultures grown with 1-BuOH, octane, phenol, and CPB were selected and systematically paired to represent dual enrichment cultures that were shaped by two carbon source classes (i.e., alcohol + alkane; alcohol + aromatic, alcohol + hydrocarbon surfactant; alkane + aromatic; alkane + hydrocarbon surfactant; or aromatic + hydrocarbon surfactant). As 1:1 (vol/vol) of two individual enrichment cultures were used in the experiment, the predicted fluoride release = (fluoride released by enrichment #1 + fluoride released by enrichment #2)/2.

Section S4. 6:2 FtSaB Biodegradation by Enrichment Cultures

No significant changes of 6:2 FtSaB were observed in no-cell controls (i.e., S-free AMS and 6:2 FtSaB only) and killed-cell controls (i.e., S-free AMS + 6:2 FtSaB + killed cells) (Figure S6A). However, trace amounts of 6:2 FtS (~0.1% of 6:2 FtSaB) were detected in the controls (Figure S6B), suggesting 6:2 FtS is present as an impurity in the 6:2 FtSaB compound (i.e., 97.1% pure) that was used in the experiments.[30] The results suggested 6:2 FtSaB remained recalcitrant. This study did not evaluate carboxylic acids as a carbon source despite the fact acetate was previously used for *Gordonia* sp. NB4-1Y and have shown 6:2 FtSaB degradation.[3] Acetate has been used as a main carbon source relatively frequent in later studies.[31-34] However, although the inclusion of carboxylic acids as an additional class of carbon source would have been informative, the direct utilization of acetate to the tricarboxylic acid cycle was assumed which potentially minimizes the induction of genes pertaining to PFAS degradation identified from previous studies.[1, 25] For instance, despite using acetate as a carbon source, activated sludge degraded 6:2 FtSaB very slowly over 109 days but was also obscured from irreversible sinks through 6:2 FtSaB adsorption.[33] From suspect-screening method, despite in level 2 confidence,[35] significantly low signals (<1% of #3736) of NIST suspect list #881 (structure of 6:2 FtSaAm [tertiary amine]) and #3825 (structure of 6:2 FtSaAm [secondary amine]) were detected at day 7 and 9 samples. Except, #3825 was not detected in the no cell control at day 0 (Figure S8B) but detected at the killed control or degradation samples which could indicate an abiotic partial degradation that have been speculative from the presence of reactive oxygen species induced from iron minerals.[4]

Section S5. Alpha Diversity, Co-occurrence Analysis, and Community Assemblage Mechanism

The shifts of microbiome composition due to enrichment of different classes of carbon sources were also reflected by the reduced values of Shannon index, an index to assess diversity based on richness and evenness of a microbial community (Table S5).[36] On the other hand, Faith's PD[37] which reflects phylogenetic distances (branch length of the tree) for diversity, increased in all conditions except when enriched with hexane or CPB. Generally, the BuOH-enriched culture was most diverse while the phenol- and CPB-enriched culture was the least diverse. The low phylogenetic diversity of hexane-enriched culture could potentially serve as the reason for diverging 6:2 FtOH and 6:2 FtS degradation results compared to the octane-enriched culture.

Co-occurrence network analysis of the enrichment populations revealed the correlations, either positive or negative, among dominant species (Figure S14 and S15). *Pseudomonas* (ASV3 and ASV8) were dominant species in EtOH- and PrOH-enriched cultures, showing negatively co-occurrence against almost all other types of bacterial populations (Figure S14). Based on the high relative abundance, the *Pseudomonas* in EtOH- and PrOH-enriched cultures could be the main active population participating in PFAS degradation. Positive correlations between *Polaromonas* and *Variovorax* were observed in BuOH- and octane- enriched cultures. In BuOH-enriched cultures, positive correlations between *Rhodococcus* and *Dyella* were observed. However, negative occurrences were observed for two most dominant populations, *Rhodococcus* and *Burkholderia,* and for *Burkholderi*a and *Dyella*.

All other enrichment communities were observed to be highly dictated by deterministic processes (i.e., tNST < 50%) (Figure S16). Overall, PrOH-, BuOH-, and octane-enriched cultures were estimated to be assembled 10~20% stochastically, which could potentially result from the higher diversity observed from previous alpha diversity indices (Table S5). Regardless, the BuOH-enriched culture exhibited the highest stochasticity, in which selective pressure from BuOH itself could be considered relatively less intensive and randomly facilitate survival of unknown PFAS degraders through mechanisms of random dispersion, random birth-death, community drifts to impact the microbial assemblage.[38]

Table S1. Target PFAS (negative mode), acronym, neutral molecular formula, and surrogate standard for analysis by QToF.

Chemical Name	Acronym	Neutral Molecular Formula[1]	Surrogate Standard
Perfluoro-n-butanoic acid	PFBA[2]	$C_4HO_2F_7$	MPFBA
Perfluoro-n-pentanoic acid	PFPeA	$C_5HO_2F_9$	M3PFPeA
Perfluoro-n-hexanoic acid	PFHxA	$C_6HO_2F_{11}$	M2PFHxA
Perfluoro-n-heptanoic acid	PFHpA	$C_7HO_2F_{13}$	M4PFHpA
Perfluoro-n-octanoic acid	PFOA	$C_8HO_2F_{15}$	M4PFOA
Perfluoro-n-nonanoic acid	PFNA	$C_9HO_2F_{17}$	M5PFNA
Perfluoro-n-decanoic acid	PFDA	$C_{10}HO_2F_{19}$	MPFDA
Perfluoro-n-undecanoic acid	PFUdA	$C_{11}HO_2F_{21}$	MPFUdA
Perfluoro-n-dodecanoic acid	PFDoA	$C_{12}HO_2F_{23}$	MPFDoA
Perfluoro-n-tridecanoic acid	PFTrDA	$C_{13}HO_2F_{25}$	MPFDoA
Perfluoro-n-tetradecanoic acid	PFTeDA	$C_{14}HO_2F_{27}$	M2PFTeDA
Perfluoro-n-hexadecanoic acid	PFHxDA	$C_{16}HO_2F_{31}$	M2PFHxDA
Perfluoropropane sulfonate	PFPrS	$C_3HO_3SF_7$	M3PFBS
Perfluorobutane sulfonate	PFBS	$C_4HO_3SF_9$	M3PFBS
Perfluoropentane sulfonate	PFPeS	$C_5HO_3SF_{11}$	M3PFBS
Perfluorohexane sulfonate	PFHxS	$C_6HO_3SF_{13}$	MPFHxS
Perfluoroheptane sulfonate	PFHpS	$C_7HO_3SF_{15}$	M3PFBS
Perfluorooctane sulfonate	PFOS	$C_8HO_3SF_{17}$	MPFOS
Perfluorononane sulfonate	PFNS	$C_9HO_3SF_{19}$	M3PFBS
Perfluorodecane sulfonate	PFDS	$C_{10}HO_3SF_{21}$	M3PFBS
Perfluorododecane sulfonate	PFDoS	$C_{12}HO_3SF_{25}$	M3PFBS
8-chloro-perfluorooctane sulfonate	Cl-PFOS	$C_8HClF_{16}SO_3$	M3PFBS
Perfluoroethylcyclohexane sulfonate	PFEtCHxS	$C_8HO_3SF_{15}$	M3PFBS
Perfluorobutane sulfonamide	FBSA	$C_4H_2O_2NSF_9$	M8FOSA
Perfluorohexane sulfonamide	FHxSA	$C_6H_2O_2NSF_{13}$	M8FOSA
Perfluorooctane sulfonamide	FOSA	$C_8H_2O_2NSF_{17}$	M8FOSA
N-methylperfluoro-1-octane sulfonamide	MeFOSA	$C_9H_4O_2NSF_{17}$	d-N-MeFOSA-M
N-ethylperfluoro-1-octane sulfonamide	EtFOSA	$C_{10}H_6O_2NSF_{17}$	d-N-EtFOSA-M
Perfluorooctane sulfonamido acetic acid	FOSAA	$C_{10}H_4O_4NSF_{17}$	d3-N-MeFOSAA
N-methylperfluorooctane sulfonamido acetic acid	MeFOSAA	$C_{11}H_6O_4NSF_{17}$	d3-N-MeFOSAA
N-ethylperfluorooctane sulfonamido acetic acid	EtFOSAA	$C_{12}H_8O_4NSF_{17}$	d5-N-EtFOSAA
4:2 fluorotelomer sulfonate	4:2 FtS	$C_6H_5O_3SF_9$	M2-4:2FTS
6:2 fluorotelomer sulfonate	6:2 FtS	$C_8H_5O_3SF_{13}$	M2-6:2FTS

8:2 fluorotelomer sulfonate	8:2 FtS	$C_{10}H_5O_3SF_{17}$	M2-8:2FTS
10:2 fluorotelomer sulfonate	10:2 FtS	$C_{12}H_5O_3SF_{21}$	M2-8:2FTS
3:3 fluorotelomer carboxylic acid	3:3 FtCA	$C_6H_5O_2F_7$	M3PFPeA
5:3 fluorotelomer carboxylic acid	5:3 FtCA	$C_8H_5O_2F_{11}$	M2PFHxA
7:3 fluorotelomer carboxylic acid	7:3 FtCA	$C_{10}H_5O_2F_{15}$	M2PFHxA
6:2 fluorotelomer carboxylic acid	6:2 FtCA	$C_8H_3O_2F_{13}$	M2PFHxA
8:2 fluorotelomer carboxylic acid	8:2 FtCA	$C_{10}H_3O_2F_{17}$	M2PFHxA
10:2 fluorotelomer carboxylic acid	10:2 FtCA	$C_{12}H_3O_2F_{21}$	M2PFHxA
2H-Perfluoro-2-octenoic acid (6:2)	6:2 FtUCA	$C_8H_2O_2F_{12}$	M6:2FTUA
2H-Perfluoro-2-decenoic acid (8:2)	8:2 FtUCA	$C_{10}H_2O_2F_{16}$	M8:2FTUA
dodecafluoro-3H-4,8-dioxanonanoate	ADONA	$C_7H_2O_4F_{12}$	MPFDA
9-chlorohexadecafluoro-3-oxanonane-1-sulfonate	9Cl-PF3ONS	$C_8HF_{16}ClSO_4$	M3PFBS
11-chloroeicosafluoro-3-oxaundecane-1-sulfonate	11Cl-PF3OUdS	$C_{10}HF_{20}ClSO_4$	M3PFBS
2,3,3,3-tetrafluoro-2-(1,1,2,2,3,3,3-heptafluoro propoxy)-propanoic acid	HFPO-DA	$C_6HF_{11}O_3$	MHFPO-DA
bis(1H,1H,2H,2H-perfluorooctyl)phosphate	6:2 diPAP	$C_{16}H_9F_{26}O_4P$	M4 8:2 diPAP
bis(1H,1H,2H,2H-perfluorodecyl)phosphate	8:2 diPAP	$C_{20}H_9F_{34}O_4P$	M4 8:2 diPAP
bis-[2-(N-ethylperfluorooctane-1-sulfonamide)ethyl]phosphate	diSAmPAP	$C_{24}H_{19}F_{34}N2O_8PS_2$	M4 8:2 diPAP

[1] $[M-H]^-$ adducts were used for quantification

[2] MRM transitions of 213 to 169 and 217 to 172 were used for quantification of PFBA and MPFBA, respectively, to reduce background.

Table S2. Target PFAS (positive mode), acronym, neutral molecular formula, and surrogate standard for analysis by QToF.

Chemical Name	Acronym	Neutral Molecular Formula[1]	Surrogate Standard
N-[3-(dimethylaminopropan-1-yl)]perfluofluoro-1-hexanesulfonamide	PFHxSaAm	$C_{11}H_{13}O_2N_2SF_{13}$	d3-Prometon
N-(carboxymethyl)-N,N, dimethyl-N-[3-(1H,1H,2H,2H-perfluoro-1-octanesulfonamido)propan-1-yl]ammonium	6:2 FtSaB	$C_{15}H_{19}O_4N_2SF_{13}$	d3-Prometon
N-[3-(perfluoro-1-hexanesulfonamido)propan-1-yl]-N,N,N-trimethylammonium	N-TAmP-FHxSA	$C_{12}H_{15}O_2SN_2F_{13}$	d3-Prometon
2-[(4,4,5,5,6,6,7,7,8,8,8-undecafluorooctyl)dimethylammonio] acetate	5:3 FtB	$C_{12}H_{14}F_{11}NO_2$	d3-Prometon
2-[(3,4,4,5,5,6,6,7,7,8,8,8-dodecafluoroooctyl)dimethylammonio] acetate	5:1:2 FtB	$C_{12}H_{13}F_{12}NO_2$	d3-Prometon

Table S3. Properties and compositions of the sandy soil used in this study.

Property	Measurement
pH	4.9
Conductivity	50 µmho/cm
Nitrate-N	3 mg/kg
Organic matter	0.05%
H_2O content	2.93 % (w/w)
Size distribution	250-500 µm (70.19%)
P	21 mg/kg
K	4 mg/kg
Ca	48 mg/kg
Mg	12 mg/kg
S	5 mg/kg
Na	5 mg/kg

Table S4. Ammonia mineral salts (AMS) media and modified S-free AMS media composition and concentrations. Prepared media was adjusted to pH 7 with HCl or NaOH. Footnotes indicate trace SO_4^{2-} residuals present in the utilized chemical stocks based on provided assays by vendor. Specific assay is unknown for chemicals without a footnote.

Purpose	AMS media	S-free AMS media	Final concentrations (mM)
Nitrogen-source	$(NH_4)_2SO_4$	NH_4Cl[a]	15.13 mM-N
Main buffer	Na_2HPO_4	Na_2HPO_4	6.1
	KH_2PO_4	KH_2PO_4[b]	3.9
Minerals and others	K_2SO_4	KCl	1.96 mM-K
	$MgSO_4·7H_2O$	$MgCl_2·6H_2O$	0.15
	$CaSO_4·2H_2O$	$CaCl_2$	0.07
	$FeSO_4·7H_2O$	$FeCl_2·4H_2O$	0.0008
	$Na_2EDTA·2H_2O$	$Na_2EDTA·2H_2O$	0.0013
	KI	KI[c]	0.0012
	$ZnSO_4·H_2O$	$ZnCl_2$	0.0021
	$MnSO_4·H_2O$	$MnCl_2·4H_2O$	0.0018
	H_3BO_3	H_3BO_3[d]	0.0016
	$CoSO_4·7H_2O$	$CoCl_2·6H_2O$[e]	0.0071
	Na_2MoO_4	Na_2MoO_4[f]	0.004

[a]: ≤0.002% SO_4^{2-} (EMD chemicals Inc., Darmstadt, Germany)

[b]: <0.0035% SO_4^{2-} (Fisher Scientific, Fair Lawn, NJ)

[c]: ≤0.005% SO_4^{2-} (EMD chemicals Inc., Darmstadt, Germany)

[d]: ≤0.01% SO_4^{2-} (EMD chemicals Inc., Darmstadt, Germany)

[e]: ≤0.01% SO_4^{2-} (EMD chemicals Inc., Darmstadt, Germany)

[f]: 0.015% SO_4^{2-} (J.T.Baker Chemical Co., Phillipsburg, NJ)

Table S5. Alpha diversity indices of original sandy soil (inoculum) and respective microbial enrichment communities. The indices depict averages and standard deviations from random 10-time sampling of respective triplicate samples at sequence depth 33,000.

Enrichment	Shannon index[a]	FaithPD[b, 37]	Observed features[c]
Soil	4.2±0.2	5.3±0.5	87±10
EtOH-enriched	2.4±0.1	7.5±1.6	35±3
PrOH-enriched	2.9±0.05	6.4±0.9	36±3
BuOH-enriched	3.4±0.05	6.8±1.1	45±4
Hexane-enriched	3.2±0.03	4.5±1.0	37±2
Octane-enriched	2.8±0.05	7.2±0.6	44±2
Phenol-enriched	2.2±0.05	6.1±0.7	30±2
CPB-enriched	2.6±0.02	3.0±0.7	14±1

[a] Shannon index = $-\sum_{i=1}^{s} p_i \ln p_i$, where s = total # of species and p_i = # of specific species i found / # of total species found

[b] $PD_i = \sum_{i \in T} I_{ij}$ x $branchlengh(T)$, where i = sample number, I_{ij} = 1 or 0 (1 if sample i has a feature from node j and 0 if sample i does not have a feature from node j), $branchlength_j(T)$ = branch length including node j in tree T

[c] total unique ASV counts

Table S6. Niche width (i.e., Levins' niche breadth) of core 34 ASVs. ASVs with high niche width values are considered generalists while specialists have low niche width values.

ASV#	Phylum	Class	Order	Family	Genus	Niche width
ASV10	Proteobacteria	Betaproteobacteria	Burkholderiales	Burkholderiaceae	*Pandoraea*	4.2
ASV15	Proteobacteria	Betaproteobacteria	Burkholderiales	Burkholderiaceae	*Pandoraea*	4.2
ASV11	Proteobacteria	Betaproteobacteria	Burkholderiales	Burkholderiaceae	*Pandoraea*	3.5
ASV7	Proteobacteria	Betaproteobacteria	Burkholderiales	Burkholderiaceae	*Pandoraea*	3.4
ASV29	Proteobacteria	Betaproteobacteria	Burkholderiales	Burkholderiaceae	*Burkholderia*	3.3
ASV18	Proteobacteria	Betaproteobacteria	Burkholderiales	Burkholderiaceae	*Burkholderia*	3.8
ASV22	Proteobacteria	Betaproteobacteria	Burkholderiales	Burkholderiaceae	*Burkholderia*	4.5
ASV37	Proteobacteria	Betaproteobacteria	Burkholderiales	Burkholderiaceae	*Burkholderia*	1.0
ASV14	Proteobacteria	Betaproteobacteria	Burkholderiales	Burkholderiaceae	*Cupriavidus*	10.2
ASV16	Proteobacteria	Betaproteobacteria	Burkholderiales	Comamonadaceae	*Polaromonas*	8.4
ASV21	Proteobacteria	Betaproteobacteria	Burkholderiales	Comamonadaceae	*Polaromonas*	9.7
ASV28	Proteobacteria	Betaproteobacteria	Burkholderiales	Comamonadaceae	*Variovorax*	5.1
ASV33	Proteobacteria	Betaproteobacteria	Burkholderiales	Comamonadaceae	*Variovorax*	5.7
ASV13	Proteobacteria	Betaproteobacteria	Burkholderiales	Comamonadaceae	*Variovorax*	4.1
ASV19	Proteobacteria	Betaproteobacteria	Burkholderiales	Comamonadaceae	*Variovorax*	4.2
ASV12	Proteobacteria	Betaproteobacteria	Burkholderiales	Comamonadaceae	*Variovorax*	4.2
ASV17	Proteobacteria	Betaproteobacteria	Burkholderiales	Comamonadaceae	*Variovorax*	4.3
ASV25	Proteobacteria	Gammaproteobacteria	Xanthomonadales	Rhodanobacteraceae	*Dyella*	11.1
ASV27	Proteobacteria	Gammaproteobacteria	Xanthomonadales	Rhodanobacteraceae	*Dyella*	12.1
ASV34	Proteobacteria	Gammaproteobacteria	Pseudomonadales	Pseudomonadaceae	*Pseudomonas*	2.0
ASV3	Proteobacteria	Gammaproteobacteria	Pseudomonadales	Pseudomonadaceae	*Pseudomonas*	6.6
ASV8	Proteobacteria	Gammaproteobacteria	Pseudomonadales	Pseudomonadaceae	*Pseudomonas*	7.8
ASV1	Proteobacteria	Gammaproteobacteria	Pseudomonadales	Pseudomonadaceae	*Pseudomonas*	3.2
ASV6	Proteobacteria	Gammaproteobacteria	Pseudomonadales	Pseudomonadaceae	*Pseudomonas*	3.6
ASV31	Bacteroidota	Flavobacteriia	Flavobacteriales	Weeksellaceae	*Elizabethkingia*	7.4
ASV24	Bacteroidota	Chitinophagia	Chitinophagales	Chitinophagaceae	*Chitinophaga*	12.9
ASV26	Bacteroidota	Chitinophagia	Chitinophagales	Chitinophagaceae	*Chitinophaga*	11.9
ASV4	Proteobacteria	Alphaproteobacteria	Sphingomonadales	Sphingomonadaceae	*Sphingomonas*	6.9
ASV9	Proteobacteria	Alphaproteobacteria	Sphingomonadales	Sphingomonadaceae	*Sphingomonas*	8.1
ASV35	Firmicutes	Bacilli	Bacillales	Paenibacillaceae	*Cohnella*	2.6
ASV20	Actinobacteriota	Actinobacteria	Mycobacteriales	Nocardiaceae	*Rhodococcus*	11.0
ASV23	Actinobacteriota	Actinobacteria	Mycobacteriales	Nocardiaceae	*Rhodococcus*	12.0
ASV5	Actinobacteriota	Actinobacteria	Mycobacteriales	Nocardiaceae	*Rhodococcus*	14.1
ASV2	Actinobacteriota	Actinobacteria	Mycobacteriales	Nocardiaceae	*Rhodococcus*	12.5

A)

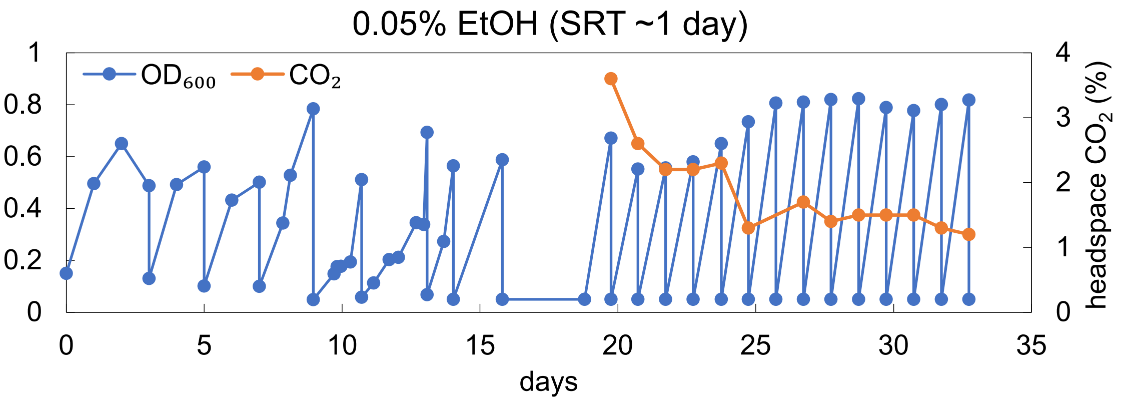
0.05% EtOH (SRT ~1 day)
OD600
CO2
OD600
headspace CO2 (%)
days

B)

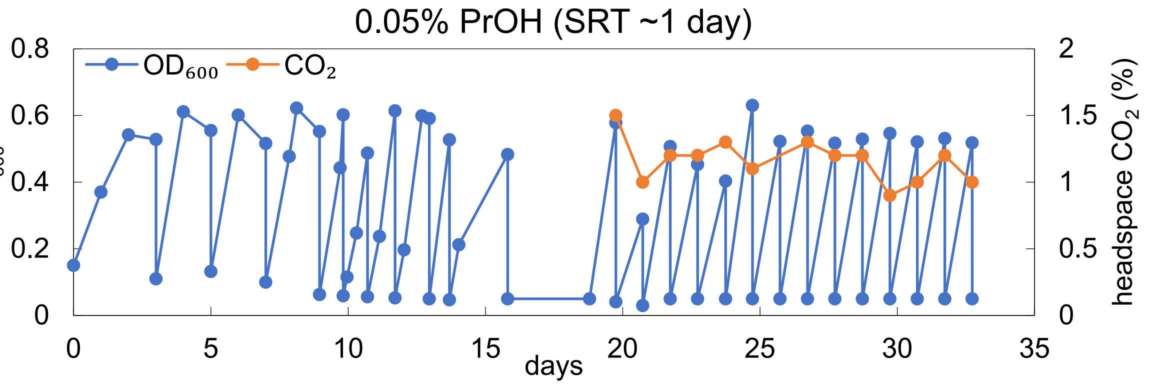
0.05% PrOH (SRT ~1 day)
OD600
CO2
OD600
headspace CO2 (%)
days

C)

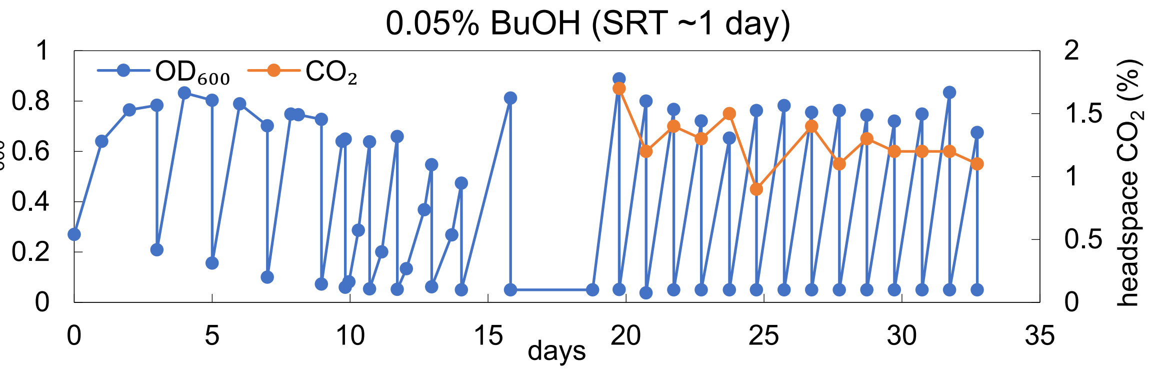
0.05% BuOH (SRT ~1 day)
OD600
CO2
OD600
headspace CO2 (%)
days

D)

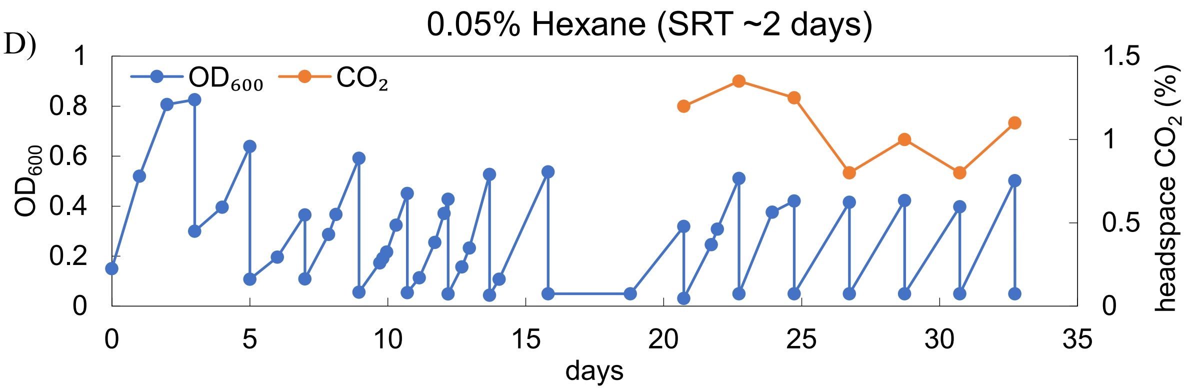
0.05% Hexane (SRT ~2 days)
OD600
CO2
OD600
headspace CO2 (%)
days

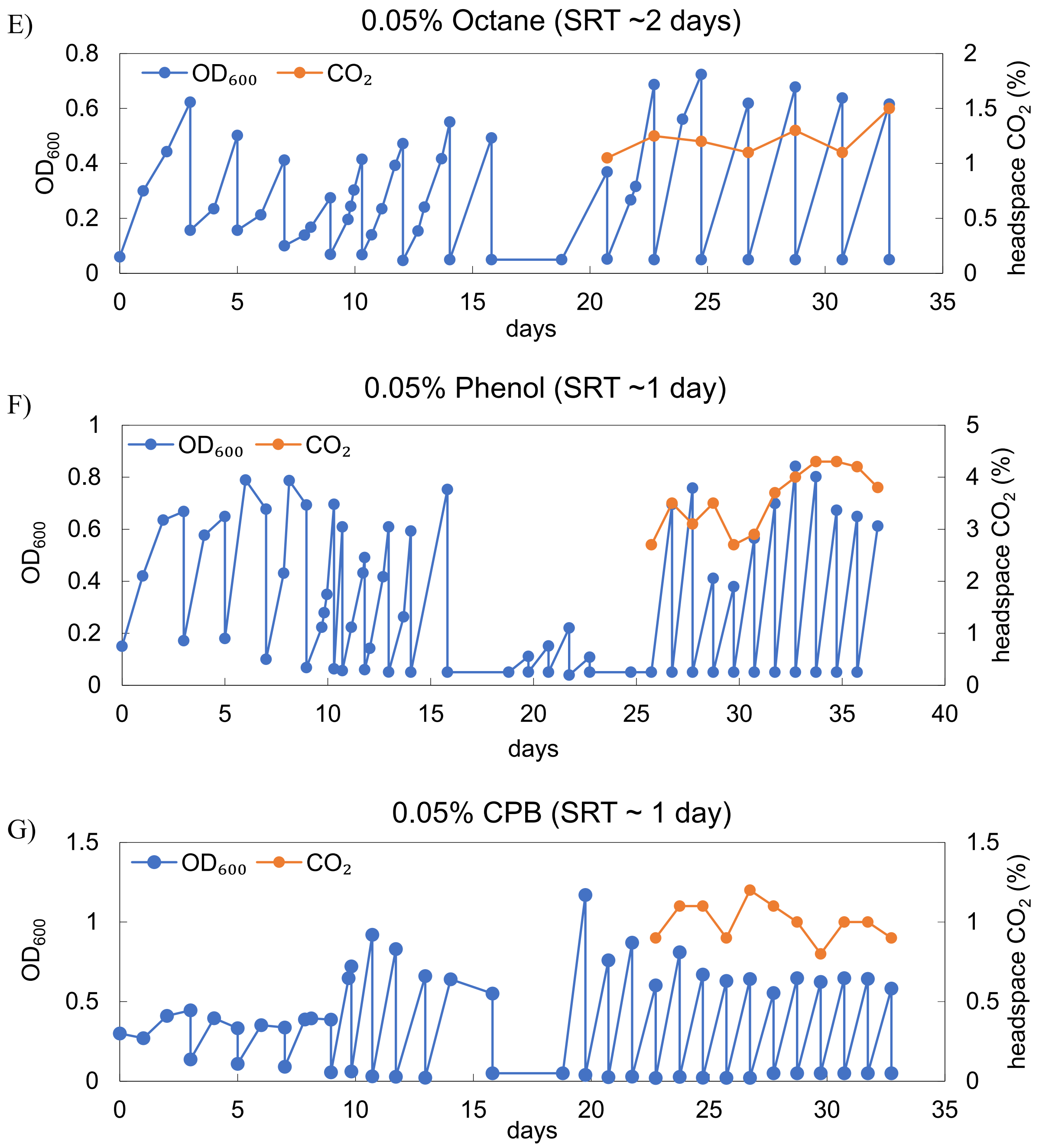

Figure S1. Time course biomass (as optical density at OD_{600}) and CO_2 concentration in sequencing batch reactors receiving difference carbon sources: A) EtOH, B) PrOH, C) BuOH, D) Hexane, E) Octane, F) Phenol, or G) CPB.

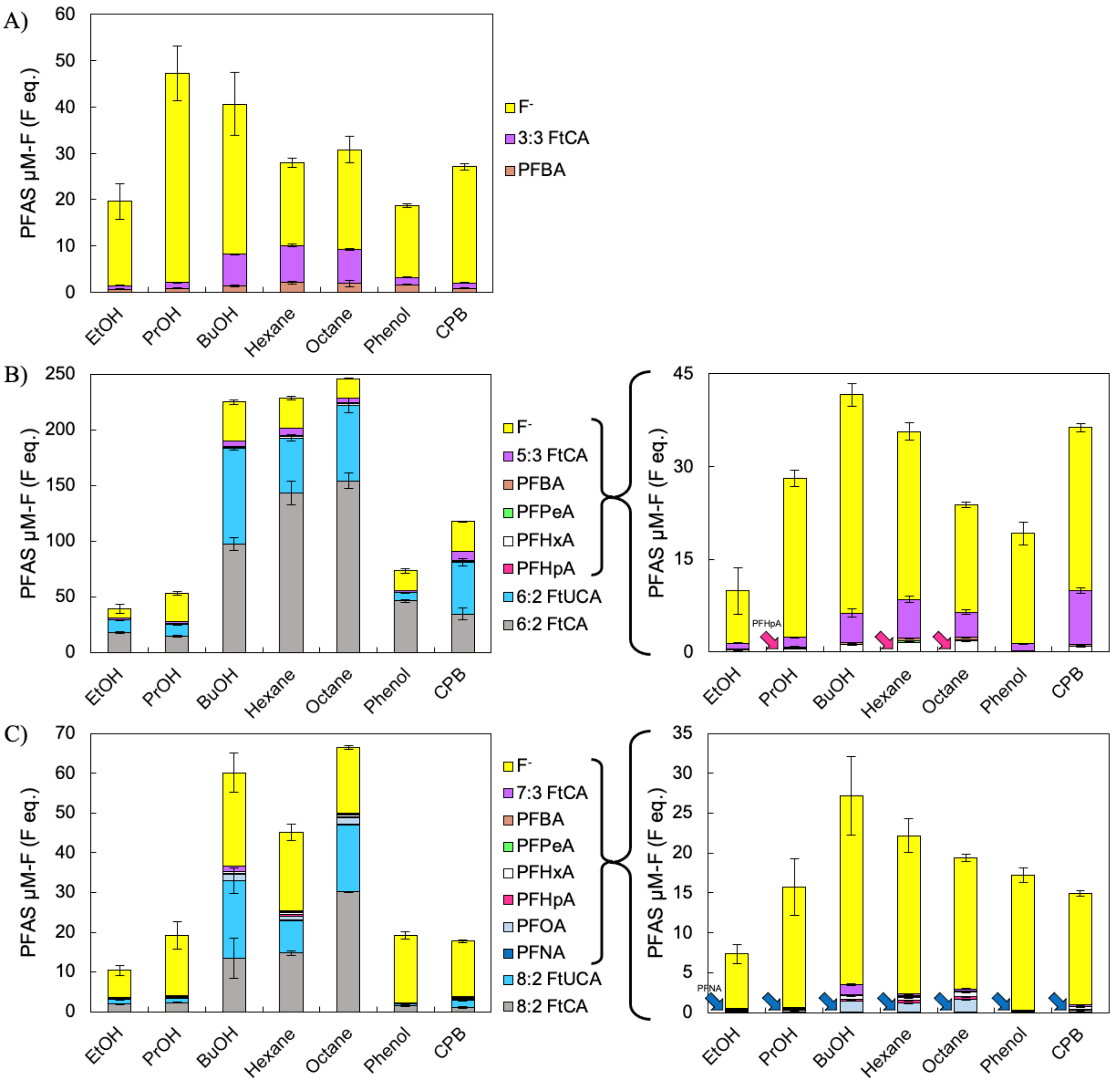

Figure S2. Degradation metabolites and fluoride levels detected from A) 4:2 FtOH, B) 6:2 FtOH, and C) 8:2 FtOH resting cell degradation experiments after three days. Fluoride equivalent (F eq) was used for all metabolites. For example, 1 mole of 3:3 FtCA = 7 moles of F eq; 1 mole of PFBA = 7 moles of F eq; 1 mole of 5:3 FtCA = 11 moles of F eq; and 1 mole of PFHxA = 11 moles of F eq. The right graph shows zoomed-in profile of measured released fluoride and FtOH degradation metabolites excluding FtCAs and FtUCAs. Bars represent ranges of duplicate sample measurements.

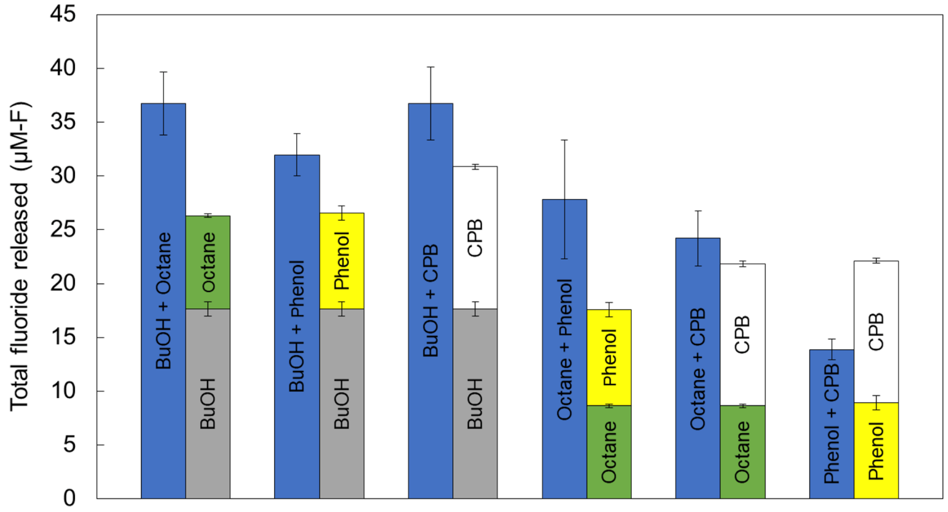

Figure S3. Fluoride released from 6:2 FtOH biodegradation by dual enrichment cultures are shown in blue. The experiments were conducted using 1:1 (vol/vol) of dual enrichments. Assuming there are no synergistic effects on the defluorination of 6:2 FtOH, the expected fluoride release by the dual cultures is 50% of the summation of fluoride release by the two individual cultures as measured in Figure 1. Bars represent ranges of duplicate sample measurements.

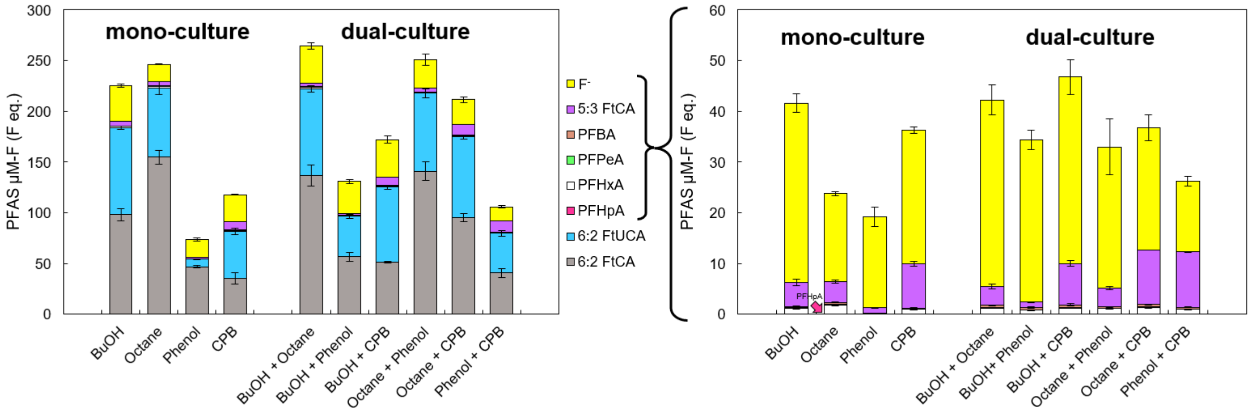

Figure S4. Degradation metabolites and fluoride detected from the mono- and dual-culture resting cell 6:2 FtOH degradation experiments. The right graph shows zoomed-in profile of 6:2 FtOH degradation metabolites of only PFHpA, PFHxA, PFPeA, PFBA, 5:3 FtCA, and the measured released fluoride. Bars represent ranges of duplicate sample measurements.

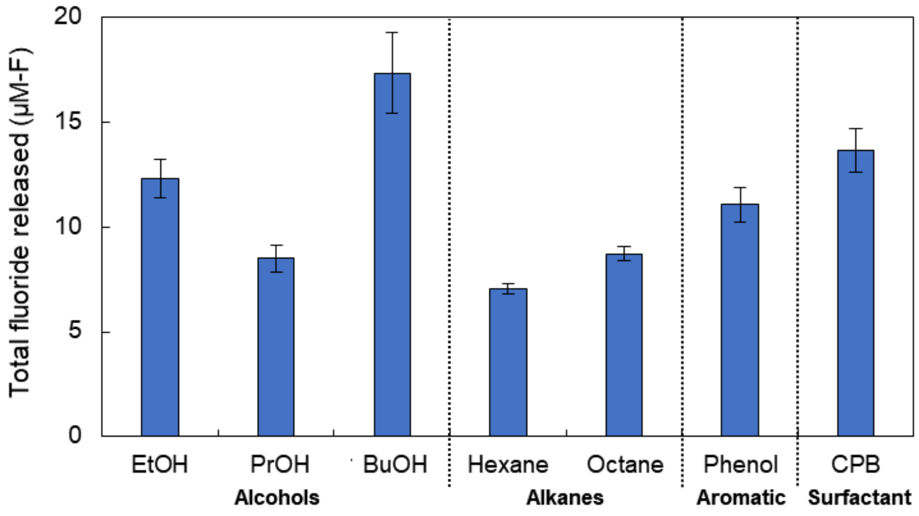

Figure S5. Total fluoride mass detected from 6:2 FtS growth experiments after eight days. The initial 6:2 FtS concentration was 20 mg/L (46.7 μmol/L = 607.1 μmole/L F eq.). Bars represent ranges of duplicate sample measurements.

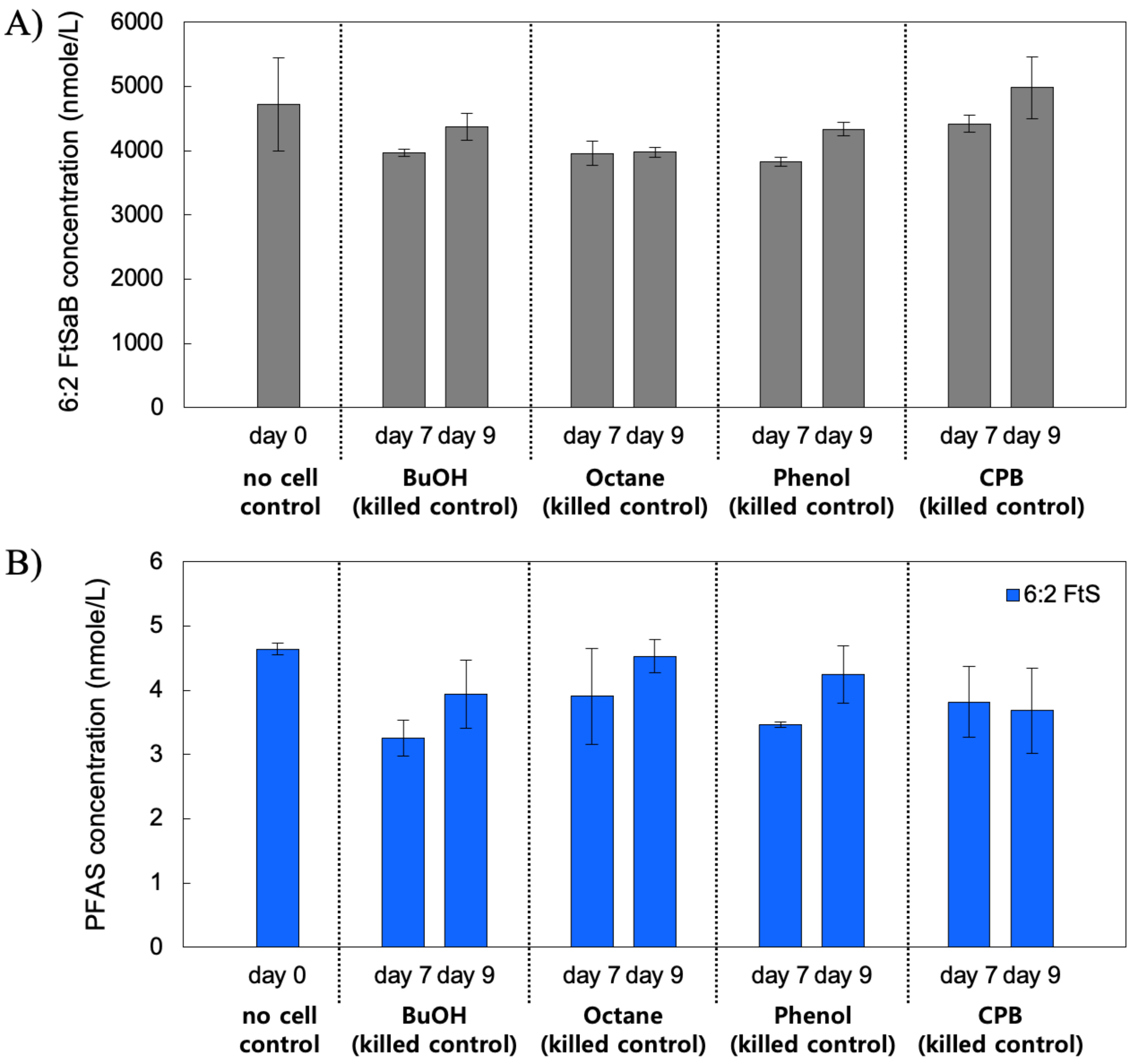

Figure S6. PFAS concentrations of the no-cell controls on Day 0 and the killed-cell controls on Day 7 and Day 9 from A) positive mode and B) negative mode target-screening methods of the 6:2 FtSaB degradation experiment samples. Bars represent ranges of duplicate sample measurements.

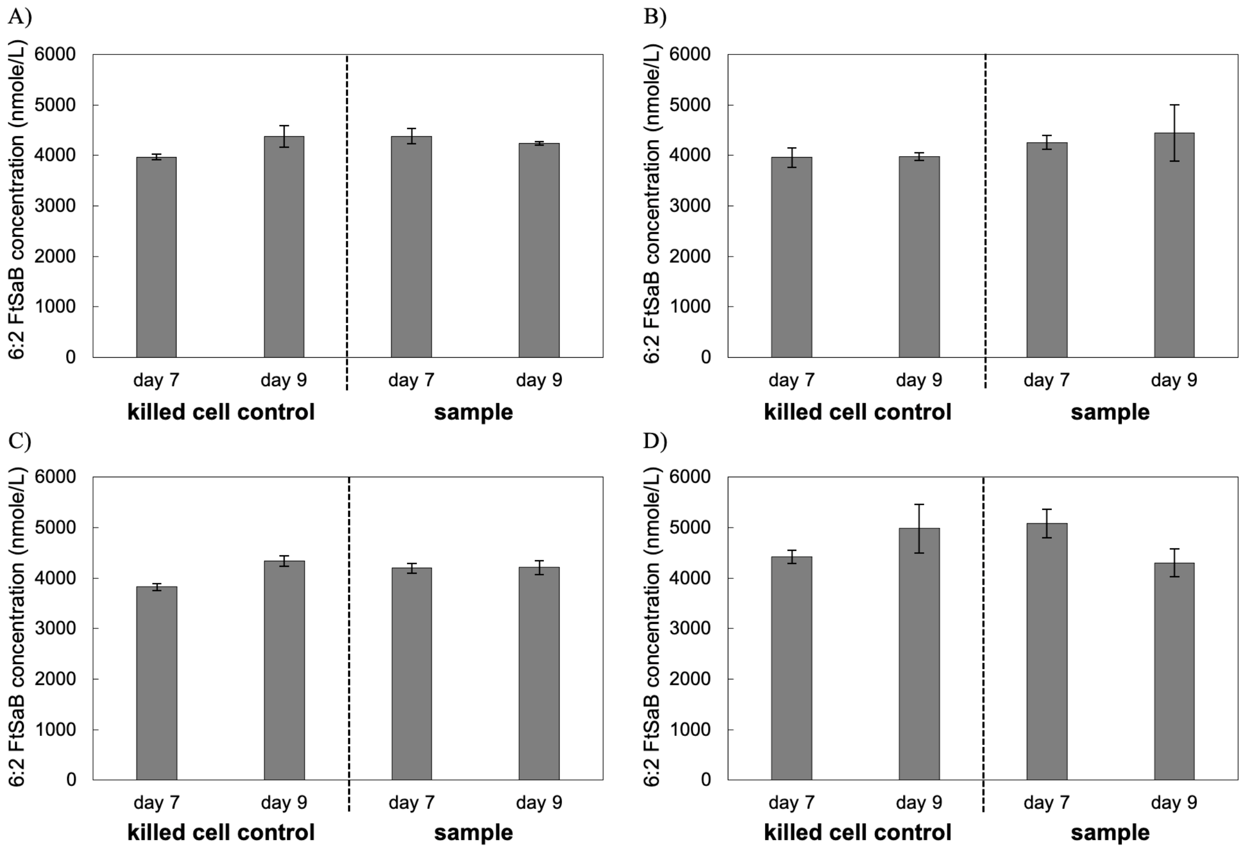

Figure S7. Concentrations of 6:2 FtSaB in killed-cell controls and samples from the 6:2 FtSaB degradation experiments on Day 7 and Day 9 by A) BuOH-, B) octane-, C) phenol-, and D) CPB-enriched cultures. Positive mode target-screening method was used. Bars represent ranges of duplicate sample measurements.

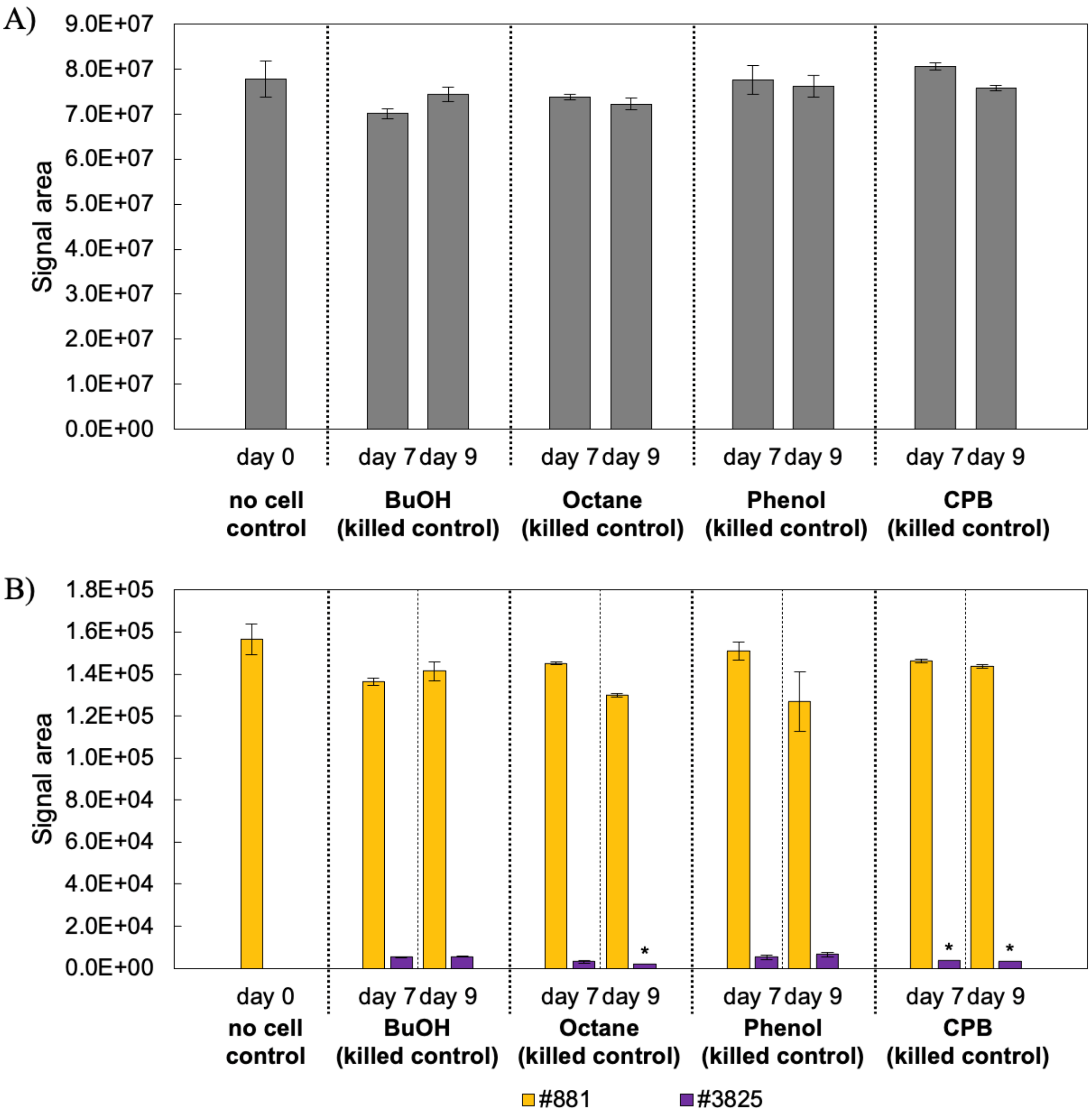

Figure S8. Suspect analysis of the no-cell controls on Day 0 and the killed-cell controls on Day 7 and Day 9 and samples of the 6:2 FtSaB degradation experiments. By using suspect-screening methods, three suspects were identified from samples. They are A) NIST suspect list #3736 (structure of 6:2 FtSaB) and B) NIST suspect list #881 (structure of 6:2 FtSaAm; tertiary amine) and #3825 (structure of 6:2 FtSaAm; secondary amine). Asterisk (*) indicates only a single signal measurement from duplicate samples. Bars represent ranges of duplicate sample measurements.

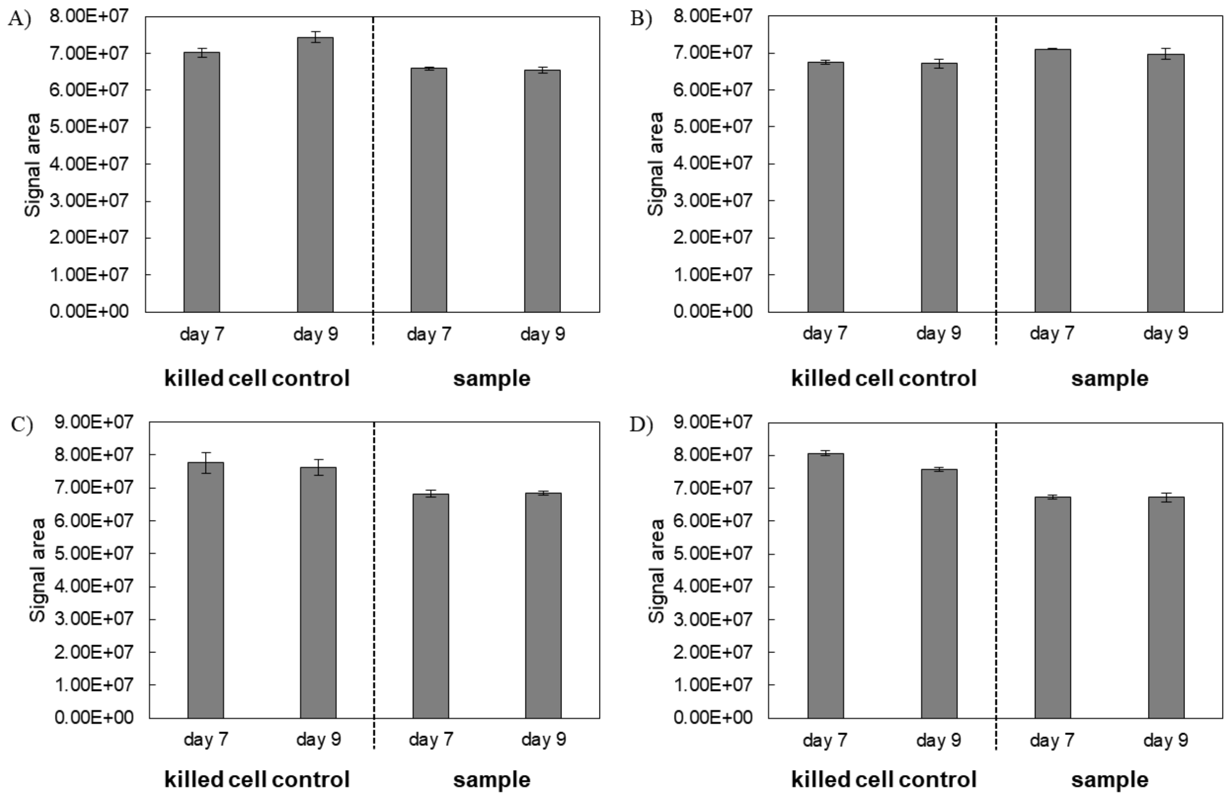

Figure S9. Comparison of NIST suspect list #3736 (structure of 6:2 FtSaB) signal areas from killed-cell controls and those from the samples of the 6:2 FtSaB degradation experiments. Samples are from A) BuOH-, B) octane-, C) phenol-, and D) CPB-enriched cultures on Day 7 and Day 9. Bars represent ranges of duplicate sample measurements.

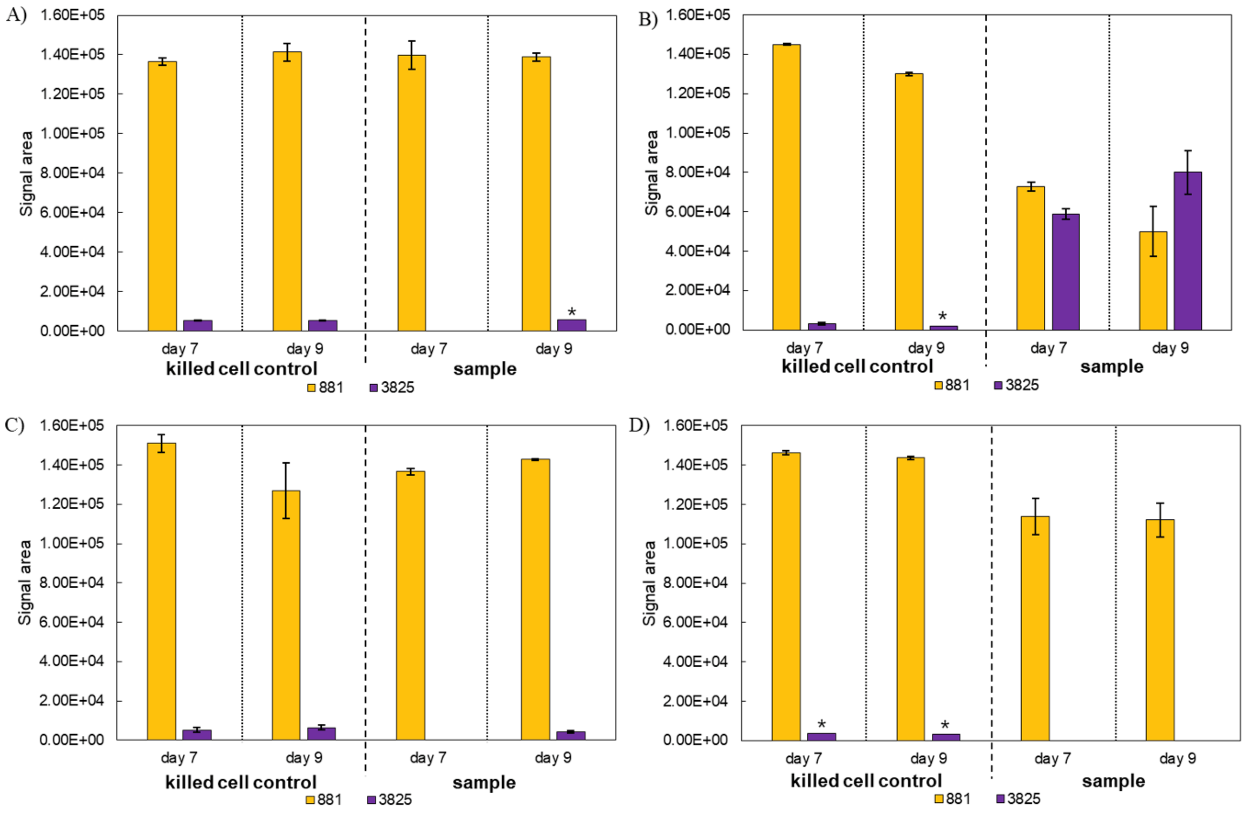

Figure S10. NIST suspect list #881 (structure of 6:2 FtSaAm; tertiary amine) and #3825 (structure of 6:2 FtSaAm; secondary amine) signal areas from killed-cell controls and samples of the 6:2 FtSaB degradation experiments. Samples are from A) BuOH-, B) octane-, C) phenol-, and D) CPB-enriched cultures on Day 7 and Day 9. Asterisk (*) indicates only a single signal measurement from duplicate samples. Bars represent ranges of duplicate sample measurements.

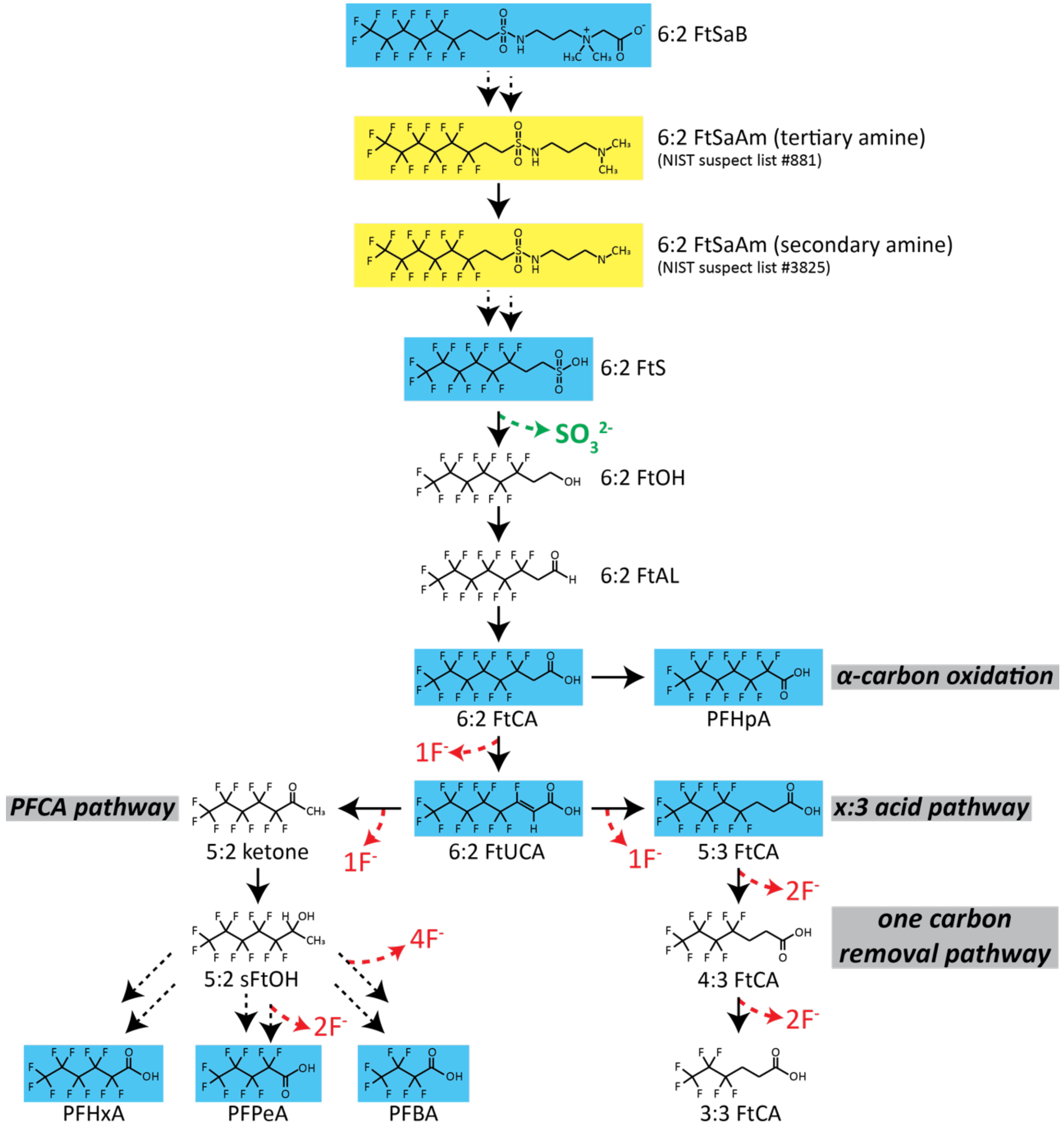

Figure S11. Proposed 6:2 FtSaB or 6:2 FtS degradation pathway adapted with references.[1-4] Compounds in blue and yellow background have been identified through target-screening and suspect-screening methods in this study, respectively from all the 6:2 FtSaB, 6:2 FtS, and 6:2 FtOH degradation experiments. Black dashed arrows indicate uncertain reactions. Green and red dashed arrows indicate the steps in which desulfonation and defluorination occur. However, to our best knowledge, defluorination genes involved in the defluorination steps of the degradation pathway remain unknown.

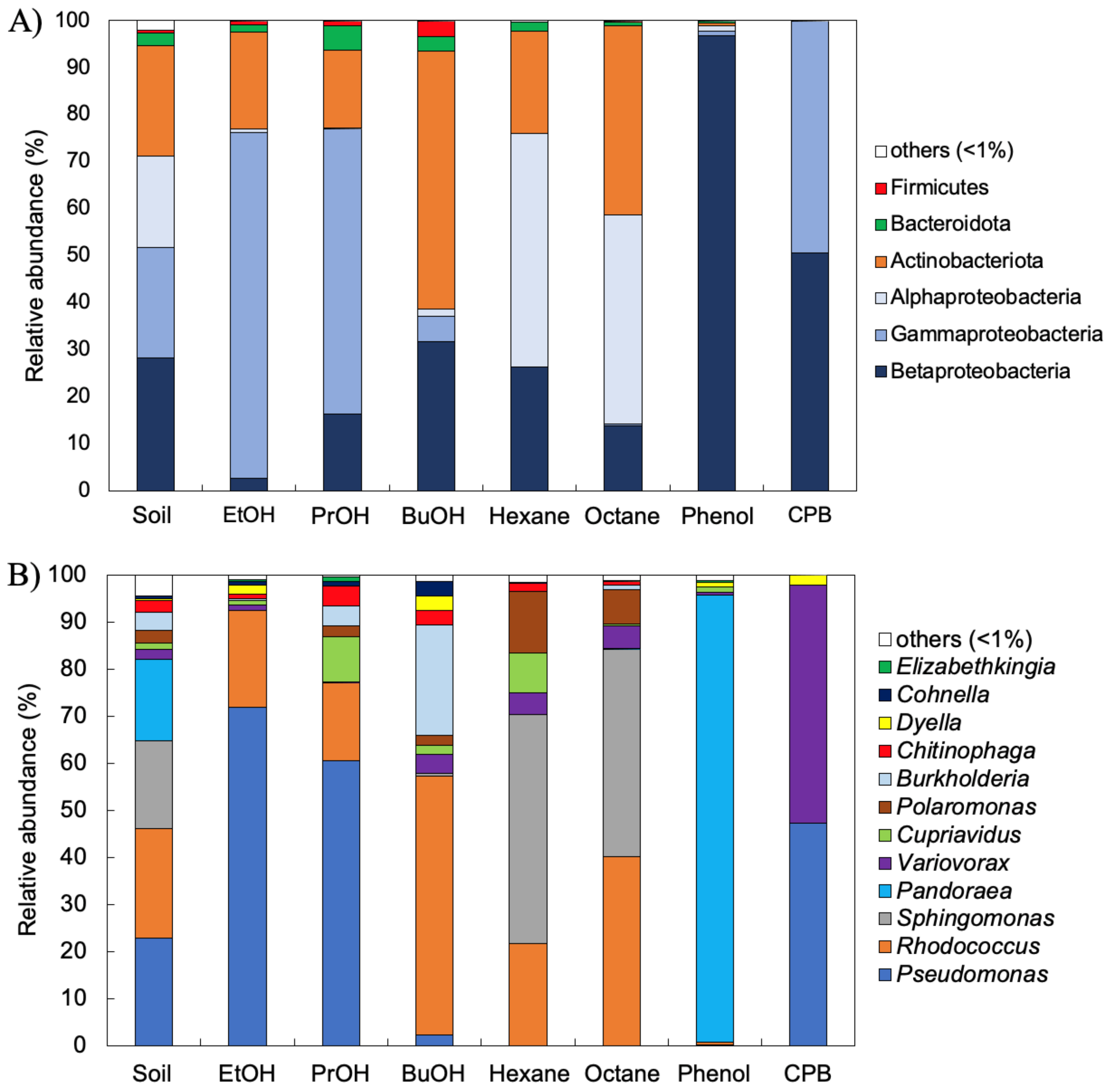

Figure S12. A) Phylum/class- and B) genus-level microbial community structure of original soil and respective enrichment cultures. The relative abundance is averaged from triplicate samples.

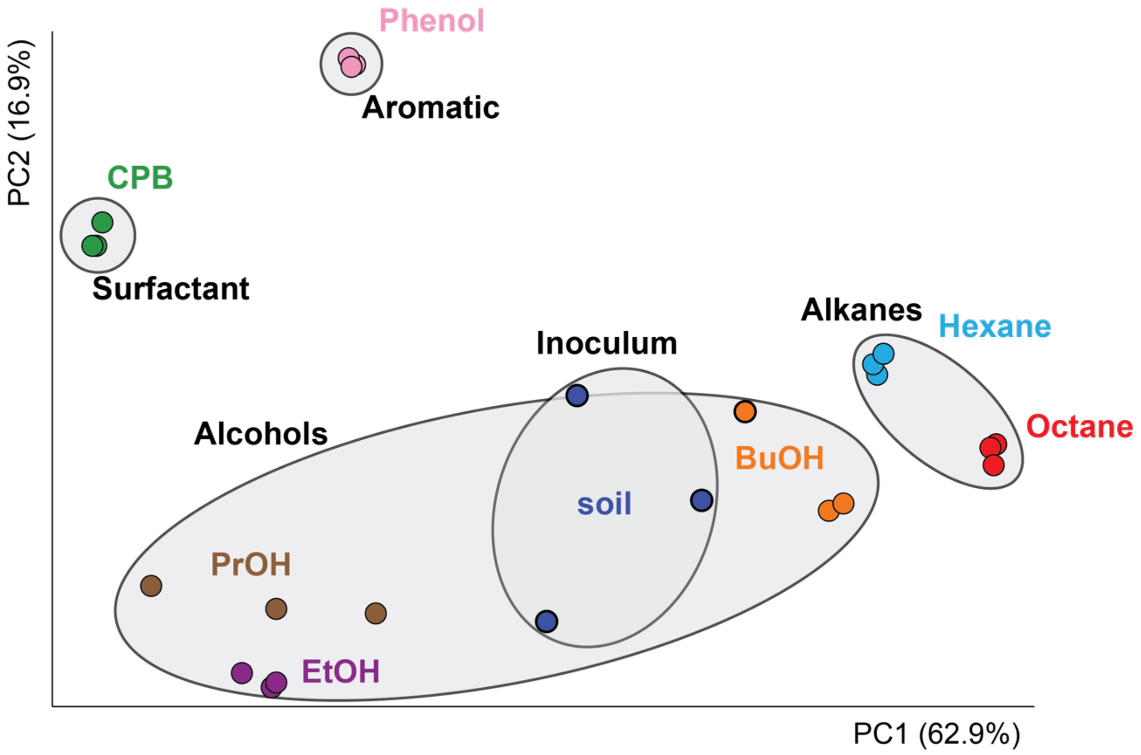

Figure S13. Principle coordinate analysis (PCoA) plot on the weighted UniFrac distance matrix referring beta-diversity of original soil and respective enrichment communities at sequence depth 33,000.

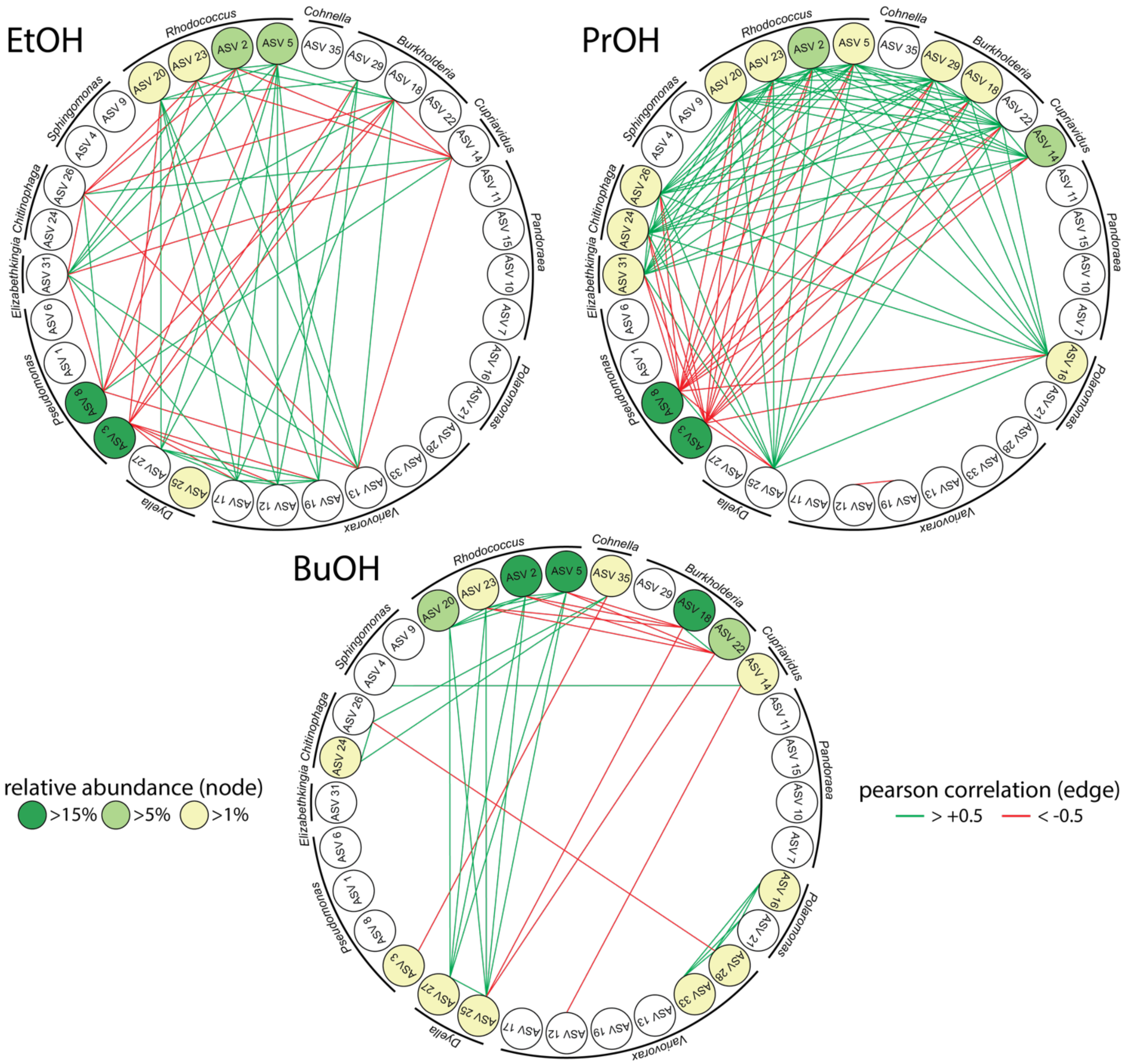

Figure S14. Co-occurrence network analysis from triplicate samples of alcohol (EtOH, PrOH, and BuOH) enrichment cultures. Only ASVs that has relative abundance >0% within all triplicate samples were considered for analysis. Nodes are highlighted in green, light green, yellow for relative abundances being >15%, >5%, and >1%, respectively. Pearson correlation coefficient values with p-value <0.05 were used for the display of edges in which green represents positive (≥0.5) and red represents negative correlations (≤-0.5).

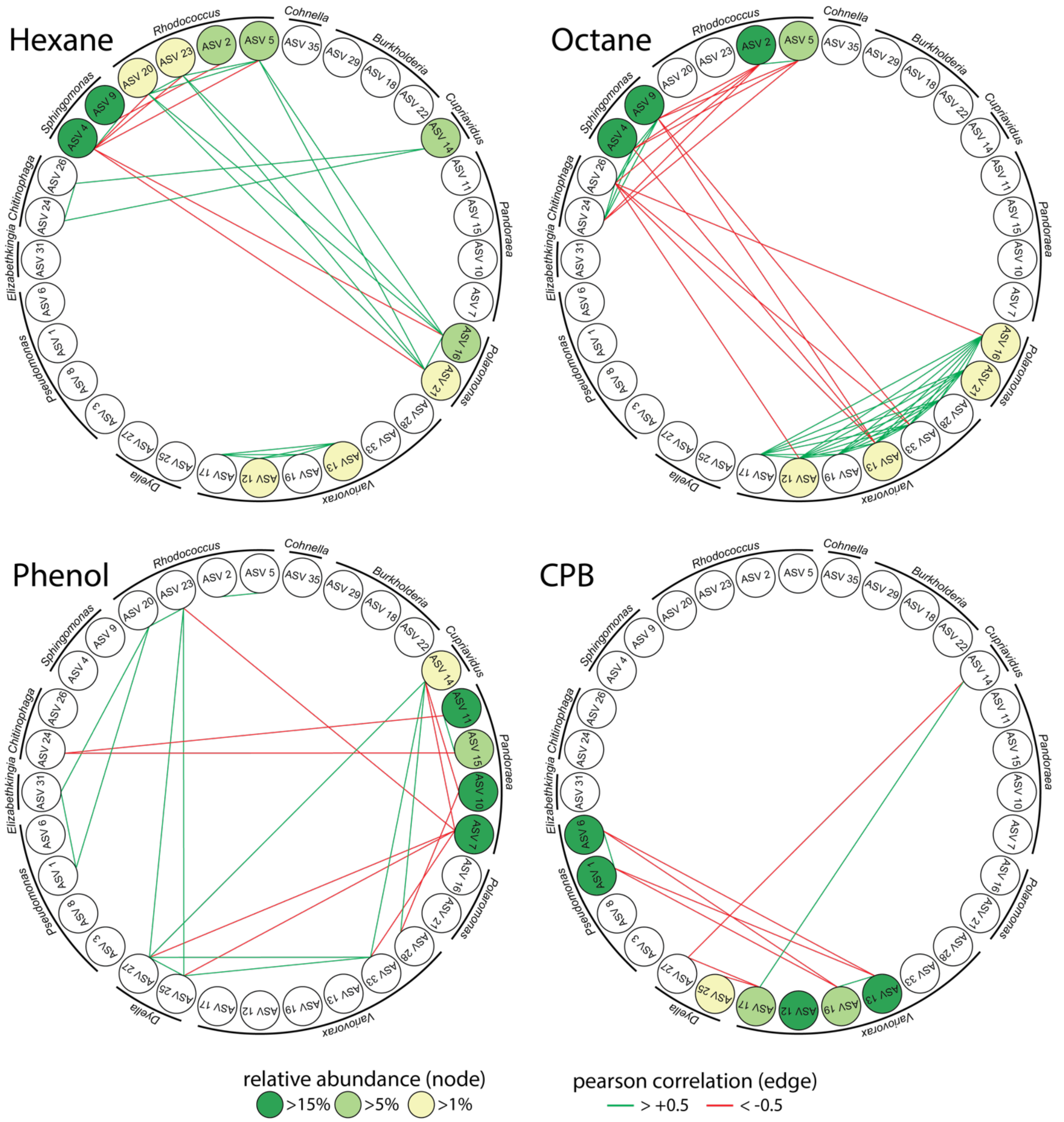

Figure S15. Co-occurrence network analysis from triplicate samples of alkane (hexane and octane), phenol, and CPB enrichment cultures. Only ASVs that has relative abundance >0% within all triplicate samples were considered for analysis. Nodes are highlighted in green, light green, yellow for relative abundances being >15%, >5%, and >1%, respectively. Pearson correlation coefficient values with p-value <0.05 were used for the display of edges in which green represents positive (≥0.5) and red represents negative correlations (≤-0.5).

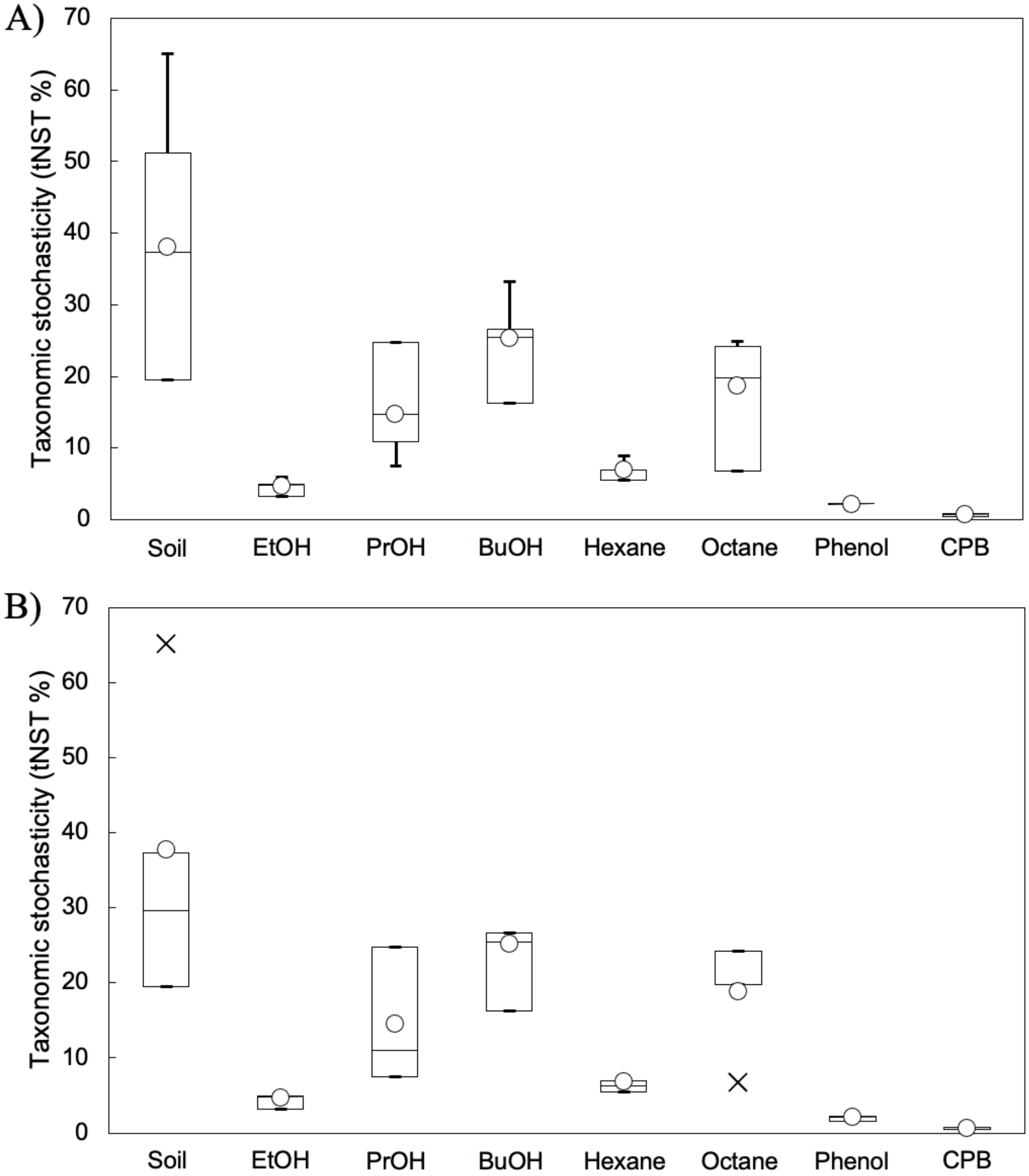

Figure S16. Box and whisker plot (○ = mean; × = outlier) of the relative contribution of the stochastic process in the microbial community assemblage. Stochasticity is represented as the estimated taxonomic normalized stochasticity ratio (tNST) index calculated from A) Jaccard (unweighted presence/absence of ASVs) and B) Bray-Curtis (relative abundance weighted presence/absence of ASVs) distance matrices by bootstrapping 1,000 times of triplicate samples. 1-tNST value represents the relative contribution of the deterministic process.

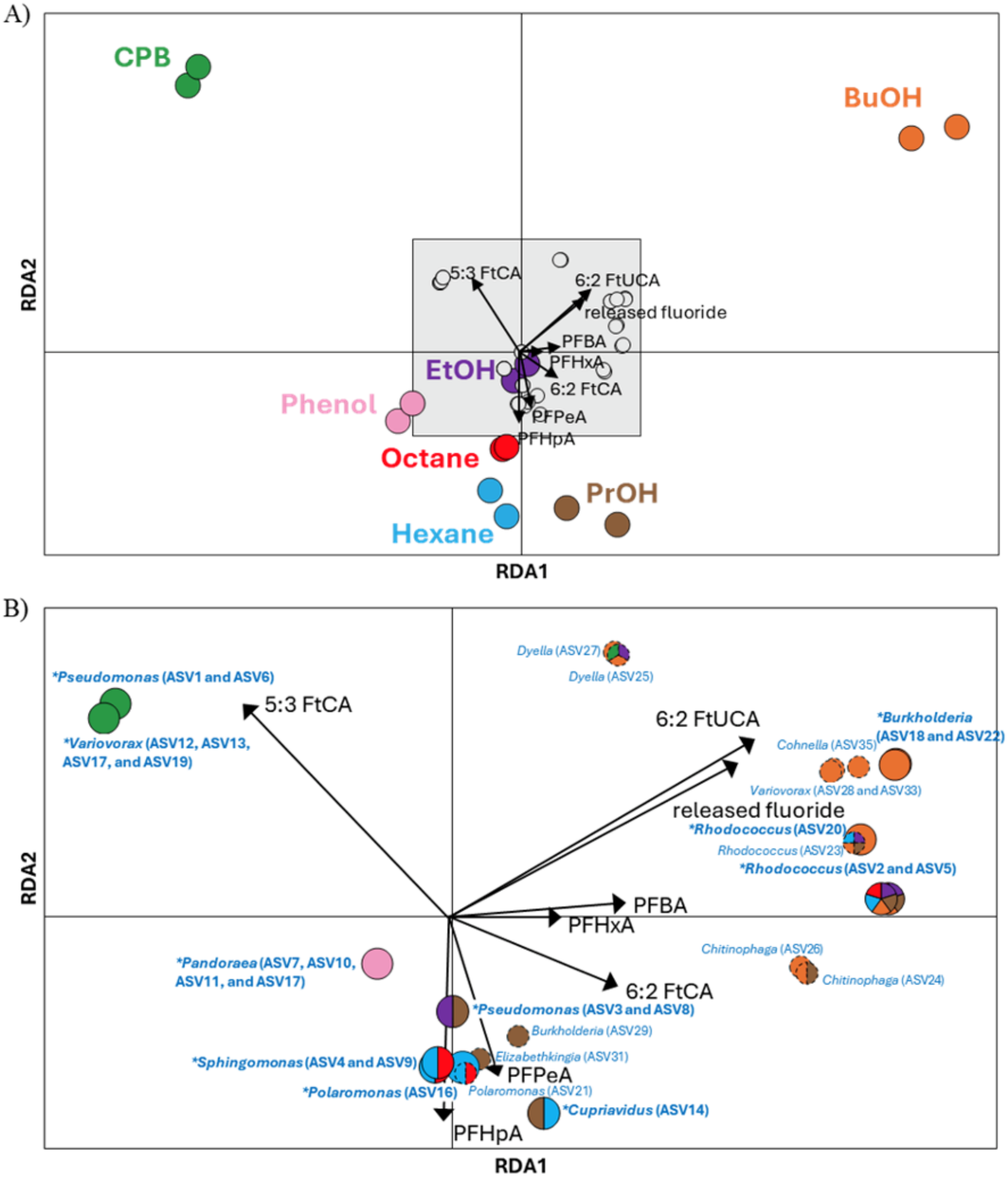

Figure S17. A) Redundancy analysis (RDA) depicting correlation between 6:2 FtOH degradation variables and ASV-level microbial composition. The gray area is zoomed-in to show B) correlation between 6:2 FtOH degradation variables and associated ASVs. Pie chart reflects the presence of the ASV within a designated enrichment culture (i.e., purple = EtOH, brown = PrOH, orange = BuOH, blue = hexane, red = octane, pink = phenol, green = CPB). Circles are large (genera are also bolded with an asterisk) if the relative abundance of the corresponding ASV is >5% within the designated enrichment culture. Circles are small and dashed if the ASV is <5% and >1%. Bold arrows within the RDA indicate variables with significance ($p<0.05$) while dashed arrows are less significant ($p>0.05$).

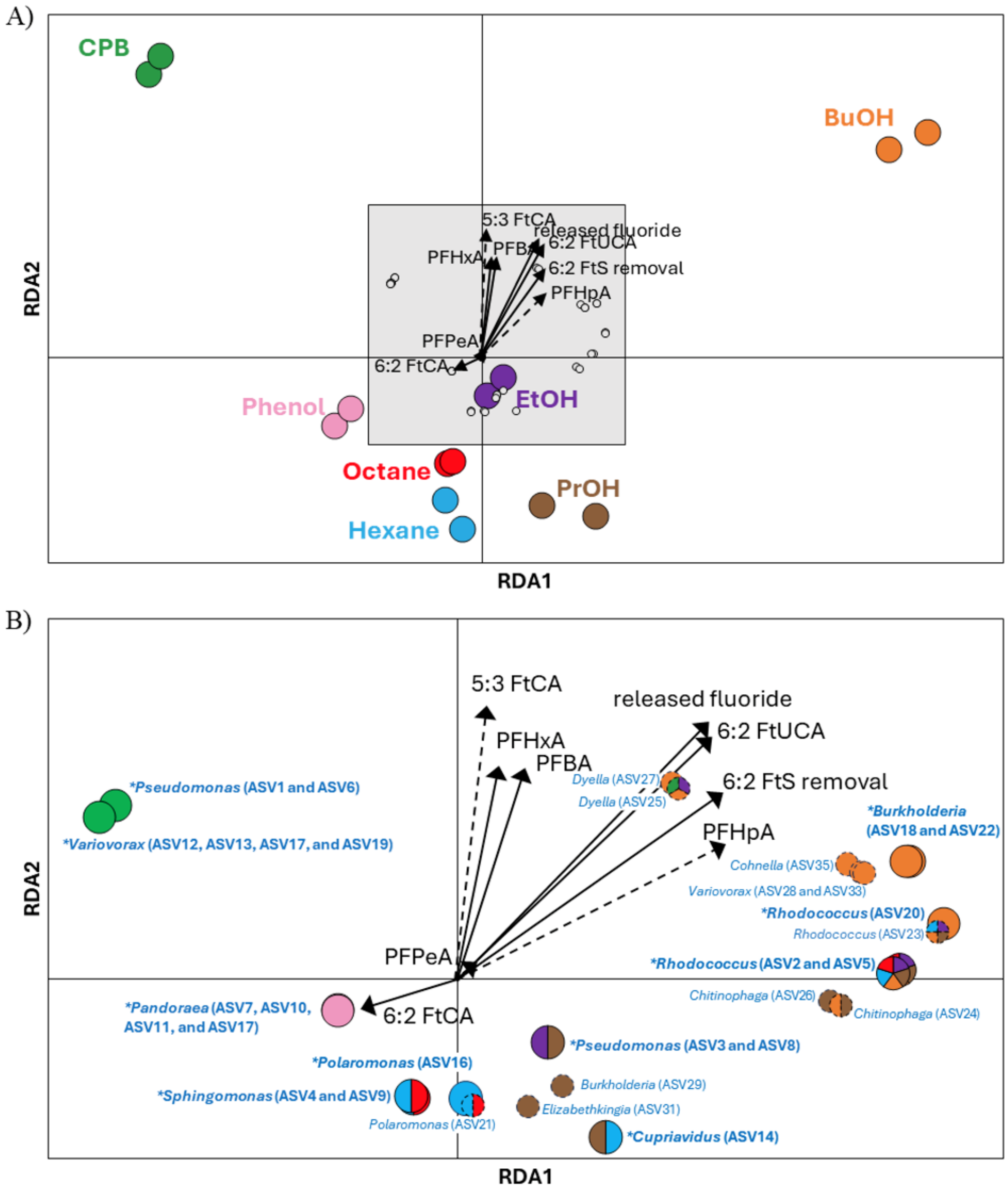

Figure S18. A) Redundancy analysis (RDA) depicting correlation between 6:2 FtS degradation variables and ASV-level microbial composition. The gray area is zoomed-in to show B) correlation between 6:2 FtS degradation variables and associated ASVs. Pie chart reflects the presence of the ASV within a designated enrichment culture (i.e., purple = EtOH, brown = PrOH, orange = BuOH, blue = hexane, red = octane, pink = phenol, green = CPB). Circles are large (genera are also bolded with an asterisk) if the relative abundance of the corresponding ASV is >5% within the designated enrichment culture. Circles are small and dashed if the ASV is <5% and >1%. Bold arrows within the RDA indicate variables with significance ($p<0.05$) while dashed arrows are less significant ($p>0.05$). The released fluoride variable for the 6:2 FtS RDA was input from the 20 mg/L 6:2 FtS degradation experiment.

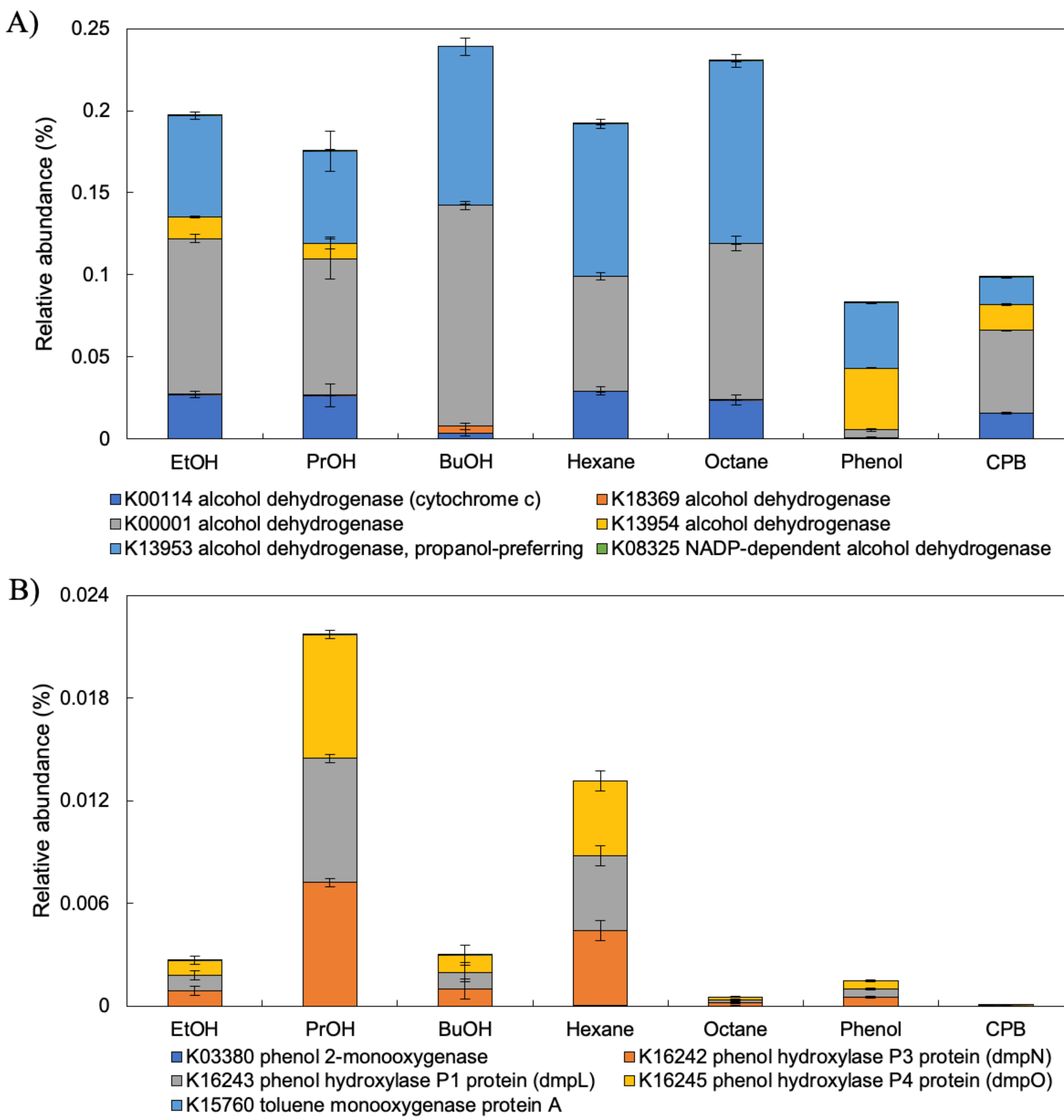

Figure S19. Relative abundance of predicted genes pertaining to A) alcohol and B) phenol/toluene oxidation.

References

(1) Yang, S.-H.; Shi, Y.; Strynar, M.; Chu, K.-H. Desulfonation and defluorination of 6:2 fluorotelomer sulfonic acid (6:2 FTSA) by Rhodococcus jostii RHA1: Carbon and sulfur sources, enzymes, and pathways. *Journal of Hazardous Materials* **2022**, *423*, 127052. DOI: https://doi.org/10.1016/j.jhazmat.2021.127052.

(2) Wang, N.; Buck, R. C.; Szostek, B.; Sulecki, L. M.; Wolstenholme, B. W. 5:3 Polyfluorinated acid aerobic biotransformation in activated sludge via novel "one-carbon removal pathways". *Chemosphere* **2012**, *87* (5), 527-534. DOI: 10.1016/j.chemosphere.2011.12.056.

(3) Shaw, D. M. J.; Munoz, G.; Bottos, E. M.; Duy, S. V.; Sauvé, S.; Liu, J.; Van Hamme, J. D. Degradation and defluorination of 6:2 fluorotelomer sulfonamidoalkyl betaine and 6:2 fluorotelomer sulfonate by Gordonia sp. strain NB4-1Y under sulfur-limiting conditions. *Science of The Total Environment* **2019**, *647*, 690-698. DOI: https://doi.org/10.1016/j.scitotenv.2018.08.012.

(4) D'Agostino, L. A.; Mabury, S. A. Aerobic biodegradation of 2 fluorotelomer sulfonamide–based aqueous film–forming foam components produces perfluoroalkyl carboxylates. *Environmental Toxicology and Chemistry* **2017**, *36* (8), 2012-2021. DOI: 10.1002/etc.3750.

(5) Kim, J.; Fuller, M. E.; Hatzinger, P. B.; Chu, K.-H. Isolation and characterization of nitroguanidine-degrading microorganisms. *Science of The Total Environment* **2024**, *912*, 169184. DOI: https://doi.org/10.1016/j.scitotenv.2023.169184.

(6) Chu, K.-H.; Alvarez-Cohen, L. Trichloroethylene degradation by methane-oxidizing cultures grown with various nitrogen sources. *Water Environment Research* **1996**, *68* (1), 76-82. DOI: https://doi.org/10.2175/106143096X127235.

(7) US National Institutes of Standards and Technology. *Suspect List of Possible Per- and Polyfluoroalkyl Substances (PFAS), https://data.nist.gov/od/id/mds2-2387*. 2021.

(8) Joseph, N. T.; Schwichtenberg, T.; Cao, D. P.; Jones, G. D.; Rodowa, A. E.; Barlaz, M. A.; Charbonnet, J. A.; Higgins, C. P.; Field, J. A.; Helbling, D. E. Target and Suspect Screening Integrated with Machine Learning to Discover Per- and Polyfluoroalkyl Substance Source Fingerprints. *Environmental Science & Technology* **2023**, *57* (38), 14351-14362. DOI: 10.1021/acs.est.3c03770.

(9) Kozich, J. J.; Westcott, S. L.; Baxter, N. T.; Highlander, S. K.; Schloss, P. D. Development of a Dual-Index Sequencing Strategy and Curation Pipeline for Analyzing Amplicon Sequence Data on the MiSeq Illumina Sequencing Platform. *Applied and Environmental Microbiology* **2013**, *79* (17), 5112-5120. DOI: doi:10.1128/AEM.01043-13.

(10) Bolyen, E.; Rideout, J. R.; Dillon, M. R.; Bokulich, N. A.; Abnet, C. C.; Al-Ghalith, G. A.; Alexander, H.; Alm, E. J.; Arumugam, M.; Asnicar, F.; et al. Reproducible, interactive, scalable and extensible microbiome data science using QIIME 2. *Nature Biotechnology* **2019**, *37* (8), 852-857. DOI: 10.1038/s41587-019-0209-9.

(11) Callahan, B. J.; McMurdie, P. J.; Rosen, M. J.; Han, A. W.; Johnson, A. J. A.; Holmes, S. P. DADA2: High-resolution sample inference from Illumina amplicon data. *Nature Methods* **2016**, *13* (7), 581-583. DOI: 10.1038/nmeth.3869.

(12) Quast, C.; Pruesse, E.; Yilmaz, P.; Gerken, J.; Schweer, T.; Yarza, P.; Peplies, J.; Glöckner, F. O. The SILVA ribosomal RNA gene database project: improved data processing and web-based tools. *Nucleic Acids Research* **2012**, *41* (D1), D590-D596. DOI: 10.1093/nar/gks1219.

(13) Camacho, C.; Coulouris, G.; Avagyan, V.; Ma, N.; Papadopoulos, J.; Bealer, K.; Madden, T. L. BLAST+: architecture and applications. *BMC Bioinformatics* **2009**, *10* (1), 421. DOI: 10.1186/1471-2105-10-421.

(14) Benton, B.; King, S.; Greenfield, S. R.; Puthuveetil, N.; Reese, A. L.; Duncan, J.; Marlow, R.; Tabron, C.; Pierola, A. E.; Yarmosh, D. A.; et al. The ATCC Genome Portal: Microbial Genome Reference Standards with Data Provenance. *Microbiology Resource Announcements* **2021**, *10* (47), e00818-00821. DOI: doi:10.1128/MRA.00818-21.

(15) Katoh, K.; Standley, D. M. MAFFT Multiple Sequence Alignment Software Version 7: Improvements in Performance and Usability. *Molecular Biology and Evolution* **2013**, *30* (4), 772-780. DOI: 10.1093/molbev/mst010.

(16) Tamura, K.; Stecher, G.; Kumar, S. MEGA11: Molecular Evolutionary Genetics Analysis Version 11. *Molecular Biology and Evolution* **2021**, *38* (7), 3022-3027. DOI: 10.1093/molbev/msab120.

(17) Saitou, N.; Nei, M. The neighbor-joining method: a new method for reconstructing phylogenetic trees. *Molecular Biology and Evolution* **1987**, *4* (4), 406-425. DOI: 10.1093/oxfordjournals.molbev.a040454.

(18) Tamura, K.; Nei, M.; Kumar, S. Prospects for inferring very large phylogenies by using the neighbor-joining method. *Proceedings of the National Academy of Sciences* **2004**, *101* (30), 11030-11035. DOI:

doi:10.1073/pnas.0404206101.
(19) Zhang, J.; Ding, Q.; Huang, J. spaa: Species association analysis. *R package version 0.2* **2016**, *2*, 33.
(20) Ning, D.; Deng, Y.; Tiedje, J. M.; Zhou, J. A general framework for quantitatively assessing ecological stochasticity. *Proceedings of the National Academy of Sciences* **2019**, *116* (34), 16892-16898. DOI: doi:10.1073/pnas.1904623116.
(21) Shannon, P.; Markiel, A.; Ozier, O.; Baliga, N. S.; Wang, J. T.; Ramage, D.; Amin, N.; Schwikowski, B.; Ideker, T. Cytoscape: A Software Environment for Integrated Models of Biomolecular Interaction Networks. *Genome Research* **2003**, *13* (11), 2498-2504. DOI: 10.1101/gr.1239303.
(22) Douglas, G. M.; Maffei, V. J.; Zaneveld, J. R.; Yurgel, S. N.; Brown, J. R.; Taylor, C. M.; Huttenhower, C.; Langille, M. G. I. PICRUSt2 for prediction of metagenome functions. *Nature Biotechnology* **2020**, *38* (6), 685-688. DOI: 10.1038/s41587-020-0548-6.
(23) Kanehisa, M.; Goto, S. KEGG: Kyoto Encyclopedia of Genes and Genomes. *Nucleic Acids Research* **2000**, *28* (1), 27-30. DOI: 10.1093/nar/28.1.27.
(24) Oksanen, J. Vegan : community ecology package. *http://vegan.r-forge.r-project.org/* **2010**.
(25) Kim, M. H.; Wang, N.; McDonald, T.; Chu, K.-H. Biodefluorination and biotransformation of fluorotelomer alcohols by two alkane-degrading Pseudomonas strains. *Biotechnology and Bioengineering* **2012**, *109* (12), 3041-3048. DOI: 10.1002/bit.24561.
(26) Kim, M. H.; Wang, N.; Chu, K.-H. 6:2 Fluorotelomer alcohol (6:2 FTOH) biodegradation by multiple microbial species under different physiological conditions. *Applied Microbiology and Biotechnology* **2014**, *98* (4), 1831-1840. DOI: 10.1007/s00253-013-5131-3.
(27) Chang, H.-L.; Alvarez-Cohen, L. Model for the Cometabolic Biodegradation of Chlorinated Organics. *Environmental Science & Technology* **1995**, *29* (9), 2357-2367. DOI: 10.1021/es00009a031.
(28) Yang, S.-H.; Shan, L.; Chu, K.-H. Root exudates enhanced 6:2 FTOH defluorination, altered metabolite profiles and shifted soil microbiome dynamics. *Journal of Hazardous Materials* **2024**, *466*, 133651. DOI: https://doi.org/10.1016/j.jhazmat.2024.133651.
(29) Lewis, M.; Kim, M.-H.; Wang, N.; Chu, K.-H. Engineering artificial communities for enhanced FTOH degradation. *Science of The Total Environment* **2016**, *572*, 935-942. DOI: https://doi.org/10.1016/j.scitotenv.2016.07.223.
(30) Barzen-Hanson, K. A.; Davis, S. E.; Kleber, M.; Field, J. A. Sorption of Fluorotelomer Sulfonates, Fluorotelomer Sulfonamido Betaines, and a Fluorotelomer Sulfonamido Amine in National Foam Aqueous Film-Forming Foam to Soil. *Environmental Science & Technology* **2017**, *51* (21), 12394-12404. DOI: 10.1021/acs.est.7b03452.
(31) Bottos, E. M.; Al-shabib, E. Y.; Shaw, D. M. J.; McAmmond, B. M.; Sharma, A.; Suchan, D. M.; Cameron, A. D. S.; Van Hamme, J. D. Transcriptomic response of Gordonia sp. strain NB4-1Y when provided with 6:2 fluorotelomer sulfonamidoalkyl betaine or 6:2 fluorotelomer sulfonate as sole sulfur source. *Biodegradation* **2020**, *31* (4), 407-422. DOI: 10.1007/s10532-020-09917-8.
(32) Fang, B.; Chen, H.; Zhou, Y.; Qiao, B.; Baqar, M.; Wang, Y.; Yao, Y.; Sun, H. Fluorotelomer betaines and sulfonic acid in aerobic wetland soil: Stability, biotransformation, and bacterial community response. *Journal of Hazardous Materials* **2024**, *477*, 135261. DOI: https://doi.org/10.1016/j.jhazmat.2024.135261.
(33) Fang, B.; Zhang, Y.; Chen, H.; Qiao, B.; Yu, H.; Zhao, M.; Gao, M.; Li, X.; Yao, Y.; Zhu, L.; et al. Stability and Biotransformation of 6:2 Fluorotelomer Sulfonic Acid, Sulfonamide Amine Oxide, and Sulfonamide Alkylbetaine in Aerobic Sludge. *Environmental Science & Technology* **2024**, *58* (5), 2446-2457. DOI: 10.1021/acs.est.3c05506.
(34) Méndez, V.; Holland, S.; Bhardwaj, S.; McDonald, J.; Khan, S.; O'Carroll, D.; Pickford, R.; Richards, S.; O'Farrell, C.; Coleman, N.; et al. Aerobic biotransformation of 6:2 fluorotelomer sulfonate by Dietzia aurantiaca J3 under sulfur-limiting conditions. *Science of The Total Environment* **2022**, *829*, 154587. DOI: https://doi.org/10.1016/j.scitotenv.2022.154587.
(35) Charbonnet, J. A.; McDonough, C. A.; Xiao, F.; Schwichtenberg, T.; Cao, D.; Kaserzon, S.; Thomas, K. V.; Dewapriya, P.; Place, B. J.; Schymanski, E. L.; et al. Communicating Confidence of Per- and Polyfluoroalkyl Substance Identification via High-Resolution Mass Spectrometry. *Environmental Science & Technology Letters* **2022**, *9* (6), 473-481. DOI: 10.1021/acs.estlett.2c00206.
(36) Shannon, C. E. A mathematical theory of communication. *The Bell System Technical Journal* **1948**, *27* (3), 379-423. DOI: 10.1002/j.1538-7305.1948.tb01338.x.
(37) Armstrong, G.; Cantrell, K.; Huang, S.; McDonald, D.; Haiminen, N.; Carrieri, A. P.; Zhu, Q.; Gonzalez, A.; McGrath, I.; Beck, K. L.; et al. Efficient computation of Faith's phylogenetic diversity with applications in characterizing microbiomes. *Genome Res* **2021**, *31* (11), 2131-2137. DOI: 10.1101/gr.275777.121 From NLM

Medline.
(38) Zhou, J.; Ning, D. Stochastic Community Assembly: Does It Matter in Microbial Ecology? *Microbiology and Molecular Biology Reviews* **2017**, *81* (4), e00002-17. DOI: doi:10.1128/mmbr.00002-17.
